# CBP/p300 and HDAC activities regulate H3K27 acetylation dynamics and zygotic genome activation in mouse preimplantation embryos

**DOI:** 10.15252/embj.2022112012

**Published:** 2022-10-10

**Authors:** Meng Wang, Zhiyuan Chen, Yi Zhang

**Affiliations:** ^1^ Howard Hughes Medical Institute Boston Children's Hospital Boston MA USA; ^2^ Program in Cellular and Molecular Medicine Boston Children's Hospital Boston MA USA; ^3^ Division of Hematology/Oncology, Department of Pediatrics Boston Children's Hospital Boston MA USA; ^4^ Department of Genetics Harvard Medical School Boston MA USA; ^5^ Harvard Stem Cell Institute Boston MA USA

**Keywords:** CBP/p300, H3K27ac, HDACs, MZT, ZGA, Chromatin, Transcription & Genomics, Development

## Abstract

Epigenome reprogramming after fertilization enables transcriptionally quiescent maternal and paternal chromatin to acquire a permissive state for subsequent zygotic genome activation (ZGA). H3K27 acetylation (H3K27ac) is a well‐established chromatin marker of active enhancers and promoters. However, reprogramming dynamics of H3K27ac during maternal‐to‐zygotic transition (MZT) in mammalian embryos are not well‐studied. By profiling the allelic landscape of H3K27ac during mouse MZT, we show that H3K27ac undergoes three waves of rapid global transitions between oocyte stage and 2‐cell stage. Notably, germinal vesicle oocyte and zygote chromatin are globally hyperacetylated, with noncanonical, broad H3K27ac domains that correlate with broad H3K4 trimethylation (H3K4me3) and open chromatin. H3K27ac marks genomic regions primed for activation including ZGA genes, retrotransposons, and active alleles of imprinted genes. We show that CBP/p300 and HDAC activities play important roles in regulating H3K27ac dynamics and are essential for preimplantation development. Specifically, CBP/p300 acetyltransferase broadly deposits H3K27ac in zygotes to induce the opening of condensed chromatin at putative enhancers and ensure proper ZGA. On the contrary, HDACs revert broad H3K27ac domains to canonical domains and safeguard ZGA by preventing premature expression of developmental genes. In conclusion, coordinated activities of CBP/p300 and HDACs during mouse MZT are essential for ZGA and preimplantation development.

## Graphical abstract

Profiling of the allelic landscape of H3K27 acetylation during mouse maternal‐to‐zygotic transition shows that H3K27ac chromatin undergoes three waves of rapid global transitions.

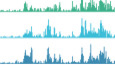

## Introduction

Mammalian life begins with the fusion of two fully differentiated gametes—the transcriptional silent metaphase II (MII) oocyte and the highly compacted sperm. During parental‐to‐zygotic transition, both maternal and paternal chromatin alleles undergo extensive epigenome reprogramming from the repressed state to a permissive and active state to confer zygotic genome activation (ZGA; Schulz & Harrison, 2019; Fu *et al*, 2020). Histone lysine acetylation modifications are well‐established active epigenetic markers (Calo & Wysocka, 2013; Shvedunova & Akhtar, 2022). The acetylation can neutralize positive charges of histones to disassociate DNA and histone interaction to generate permissive chromatin (Chen *et al*, 2022). Histone acetylation can be recognized by transcription coactivators to facilitate preinitiation complex (PIC) assembly, transcription initiation, and elongation (Narita *et al*, 2021). Particularly, histone H3 lysine 27 acetylation (H3K27ac) has been widely recognized as a marker for active enhancers and promoters (Creyghton *et al*, 2010). However, the relationship between H3K27ac and ZGA in mammalian early embryos has not been fully studied.

The developments of ultra‐low‐input chromatin immunoprecipitation (ChIP; Brind'Amour *et al*, 2015) and immunocleavage (ChIC; Skene & Henikoff, 2017) sequencing methods have enabled the study of the dynamic reprogramming of different histone modifications and histone variants during mammalian maternal‐to‐zygotic transition (MZT), which revealed diverse reprogramming patterns for H3K4me3 (Dahl *et al*, 2016; Zhang *et al*, 2016), H3K27me3 (Liu *et al*, 2016; Zheng *et al*, 2016), H3K9me3 (Wang *et al*, 2018), H3K36me3 (Xu *et al*, 2019), H2AK119ub1 (Chen *et al*, 2021; Mei *et al*, 2021), H3.3 (Ishiuchi *et al*, 2020) and H2A.Z (Liu *et al*, 2022). The study of H3K27ac during zebrafish MZT has revealed distinct dynamics between promoters and distal regions (Zhang *et al*, 2018; Chan *et al*, 2019; Miao *et al*, 2022). A previous study focused on H3K4me3 in mouse early embryos has profiled H3K27ac with limited stages, missing the important zygote stage (Dahl *et al*, 2016). Since H3K27ac is turned over rapidly (Weinert *et al*, 2018), a study with consecutive key stages during MZT is needed to reveal the dynamics of H3K27ac in mouse preimplantation embryos. In addition, although reporter assays have suggested that major ZGA requires enhancer activity (Schultz, 2002), whether endogenous ZGA genes are regulated by enhancers and whether H3K27ac is indicative of enhancer activities during ZGA remains unknown.

The H3K27ac is deposited by the two paralogue histone acetyltransferases (HATs) CBP and p300, which function as transcription coactivators that mediate the binding of transcription factors (TF) and RNA polymerase II (pol II; Weinert *et al*, 2018). CBP/p300‐mediated acetylation is sufficient to activate gene expression (Miao *et al*, 2022). Targeting CBP/p300 to promoters or enhancers by CRISPR/dCas9 can activate the downstream genes (Hilton *et al*, 2015). The HAT activity of CBP/p300 is critical for the activation of enhancers, as the binding of CBP/p300 per se cannot activate enhancers (Narita *et al*, 2021). The steady state of histone acetylation levels is regulated by the balanced activities of HATs and histone deacetylases (HDACs). The mammalian genomes encode 11 classical HDACs (HDAC1–11; Haberland *et al*, 2009). Depletion of HDAC1/2 results in early embryonic lethality (Ma & Schultz, 2008, 2016; Zhao *et al*, 2020). However, the role of CBP/p300 and HDACs in modulating mammalian MZT and how their activities contribute to ZGA remain largely unknown.

In this study, by profiling the allelic H3K27ac landscape in mouse oocytes and early embryos, we unexpectedly observed noncanonical large broad acetylation domains in germinal vesicle (GV) oocytes and zygotes. The acetylation landscape undergoes three major waves of global transition during MZT: (i) hyperacetylation in GV oocytes to hypoacetylation in MII oocytes; (ii) hypoacetylation in MII oocytes to hyperacetylation in zygotes; (iii) hyperacetylation in zygotes to canonical acetylation patterns (narrow peaks) in early 2‐cells. The second and third waves of global transition indicate rapid turn‐over of acetylation after fertilization, which coincides with CBP/p300 and HDACs activities, respectively. We show that CBP/p300 acetyltransferase activity is required for *de novo* establishment of the hyperacetylation state in zygotes and minor ZGA. Furthermore, CBP/p300 activity is required for opening chromatin at putative enhancers for major ZGA genes. By contrast, HDACs activities are essential for the deacetylation from zygote to early 2‐cell (i.e., the third wave transition) and were critical for preventing premature expression of developmental genes as well as for ensuring normal ZGA. Our study thus not only revealed the H3K27ac dynamics during MZT but also established a critical function of the CBP/p300 and HDACs activities in mouse ZGA and preimplantation development.

## Results

### Dynamic landscape of H3K27ac during mouse MZT

**Figure 1:**
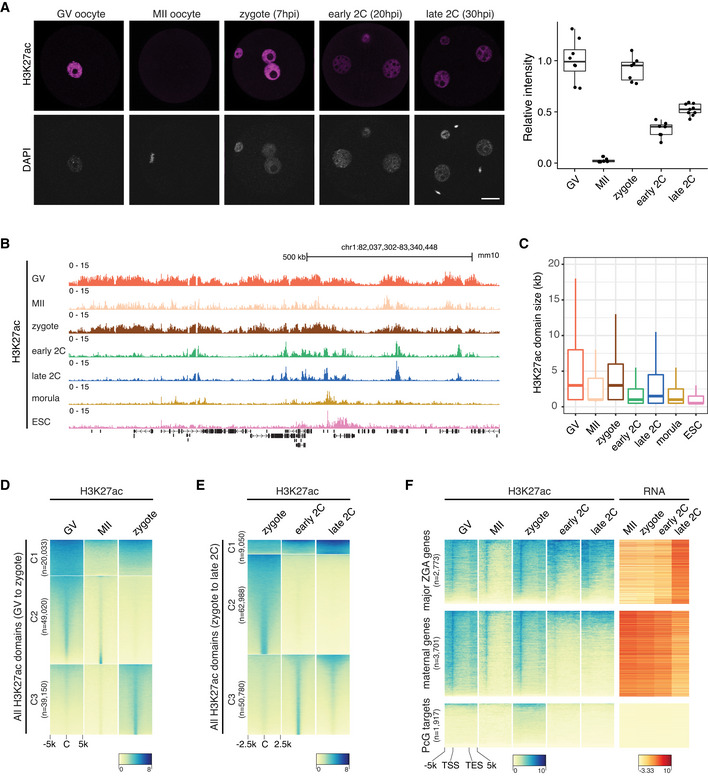
Dynamic landscape of H3K27ac in mouse oocytes and early embryos Immunostaining of H3K27ac in mouse germinal vesicle (GV) oocytes (*n* = 8), metaphase II (MII) oocytes (*n* = 7), zygotes (*n* = 7), early 2‐cell (2C; *n* = 7) and late 2C (*n* = 9) embryos. Right panel is the quantification of H3K27ac immunostaining relative signal intensities at different stages. Scale bar: 20 μm.Genome browser view showing the landscape of H3K27ac in oocytes and early embryos.H3K27ac domain size distribution at different stages.Dynamic changes of H3K27ac from GV oocytes to zygotes. All the H3K27ac domains from GV to zygote were classified into three clusters (C1–C3) using k‐means clustering. C—domain center.Dynamic changes of H3K27ac from zygotes (before ZGA) to late 2‐cell embryos (major ZGA). All the H3K27ac domains from zygotes to late 2‐cell embryos were classified into three clusters (C1–C3) using k‐means clustering.Enrichment of H3K27ac at major ZGA genes, maternal decay genes and Polycomb group (PcG) targets at different stages. TSS—transcription start site; TES—transcription end site. Data information: For boxplots in (A) and (C), the central band represents the median. The lower and upper edges of the box represent the first and third quartiles, respectively. The whiskers of the boxplot extend to 1.5 times interquartile range (IQR).

To study the global reprogramming dynamics of H3K27ac in mouse oocytes and early embryos, we first performed H3K27ac whole‐mount immunostaining (Fig 1A). The results revealed a dramatic decrease in H3K27ac level from GV to MII oocytes, which is in line with a previous study showing global deacetylation of histone H3 lysine 14 and histone H4 lysine 12 during oocyte meiosis (Kim *et al*, 2003). Intriguingly, the H3K27ac level was restored quickly in zygotes, to a level similar to that in GV oocytes, indicating *de novo* acetylation occurs shortly after fertilization. The acetylation level decreased again at early 2‐cell stage. Those results indicate three waves of global turn‐over of H3K27ac levels during MZT.

**Figure EV1:**
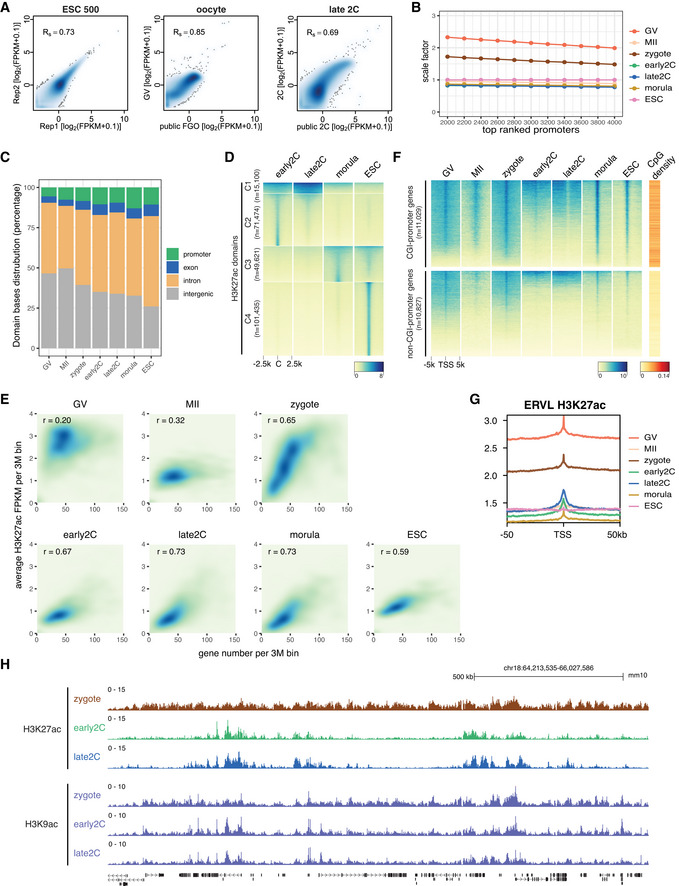
Dynamics of H3K27ac in mouse oocytes and early embryos Spearman correlation of H3K27ac CUT&RUN replicates and correlation with available public data. The public H3K27ac data were generated using the μChIP–seq method (Dahl *et al*, 2016). GV—germinal vesicle oocyte; FGO—fully grown oocyte; 2C—2‐cell embryo.Scale factors for H3K27ac FPKM at different stages. The scale factors at top 3,000 promoters were used.H3K27ac domain bases distribution at promoter, exon, intron, and intergenic regions for each stage.Dynamic changes of H3K27ac from early 2‐cell to morula stage and ESC. C: domain center.Correlations between gene density and H3K27ac signals for each stage.H3K27ac signal enrichment around TSS of genes with CGI or non‐CGI promoters. CGI: CpG island.Enrichment of H3K27ac at ERVL retrotransposons at different stages.Genome browser view showing different dynamics of H3K27ac and H3K9ac from zygotes to late 2‐cell embryos.

The rapid reprogramming of acetylation and deacetylation during MZT prompted us to examine the detailed dynamic landscapes of H3K27ac in mouse oocytes and early embryos. To this end, we tested the ultra‐low‐input CUT&RUN method using as few as 500 mouse embryonic stem cells (mESCs) and the two replicates showed high correlation (Fig EV1A). Then, we employed the CUT&RUN to profile H3K27ac in GV oocytes, MII oocytes, zygotes, early 2‐cell, late 2‐cell, and morula stage embryos (Fig 1B). Our data showed high correlation with existing public data (Dahl *et al*, 2016) of oocytes and 2‐cell embryos, which were generated by a different profiling method with a different antibody (Fig EV1A). To make the H3K27ac signals (FPKM) comparable for different patterns in different stages, we normalized the FPKM values based on the promoter signals with high H3K27ac levels (Dahl *et al*, 2016; Fig EV1B, see Materials and Methods). Unexpectedly, the landscape of H3K27ac exhibited noncanonical large broad H3K27ac domains in GV oocytes and zygotes (Fig 1B and C), indicating chromatin hyperacetylation in GV oocytes and zygotes. By contrast, the MII oocytes had very sparse signals. After the first cell cleavage, the H3K27ac signals were reprogrammed to a canonical pattern with narrow peaks at early 2‐cell stage. The late 2‐cell (major ZGA) H3K27ac signal distribution generally resembled that of early 2‐cell (before major ZGA). From late 2‐cell to morula stage, the H3K27ac signals resolved to more sharp peaks, as in the ESCs. The domain size distribution in different stages was consistent with the immunostaining signal intensities (Fig 1A and C), and H3K27ac occupied both genic and intergenic regions (Fig EV1C).

The above H3K27ac profiling revealed the dynamics of three major waves of H3K27ac transition during MZT (Figs 1D and E, and EV1D). The first wave takes place from the hyperacetylated GV oocytes to the hypoacetylated MII oocytes, where all the broad acetylation domains disappear (Fig 1D, cluster C1) while a few narrow domains remain (Fig 1D, cluster C2). The second wave is the regaining of broad acetylation (Fig 1D, cluster C1) and narrow acetylation domains (Fig 1D, cluster C3) in zygotes. The third wave occurs from the hyperacetylated zygotes to the early 2‐cell embryos, where most broad domains in zygotes are deacetylated (Fig 1E, cluster C2) and canonical acetylation peaks are *de novo* established (Fig 1E, cluster C3), while a few broad domains are maintained from zygotes to early 2‐cell and late 2‐cell embryos (Fig 1E, cluster C1).

### ZGA gene promoters are primed with H3K27ac

Although the H3K27ac is a well‐established chromatin marker of active transcription (Shvedunova & Akhtar, 2022), there is almost no active transcription in the hyperacetylated zygotes. This observation suggests a transcription independent *de novo* deposition of H3K27ac in zygotes. Nevertheless, we observed a strong correlation between gene density and H3K27ac level in zygotes, as well as in 2‐cell, morula embryos and ESCs (Fig EV1E), indicating H3K27ac is enriched in gene dense regions while depleted in gene deserts. In GV oocytes, there is no such correlation between gene density and H3K27ac level (Fig EV1E), indicating different regulations of H3K27ac deposition in oocytes and zygotes. The hyperacetylation of gene dense regions in zygotes prompted us to check H3K27ac levels for different groups of genes. In general, most genes, especially the ones with CpG island (CGI) promoters, showed enrichment of H3K27ac at their promoters in GV oocytes and zygotes (Fig EV1F). Intriguingly, the major ZGA genes, which are highly expressed at late 2‐cell, showed H3K27ac enrichment at their transcriptional start site (TSS) regions in zygotes and early 2‐cell embryos (Fig 1F). Even mild H3K27ac enrichment can be observed in GV and MII oocytes for the major ZGA genes. For the maternal genes, H3K27ac enrichment in GV oocytes was the highest (Fig 1F), but the signals decreased in MII oocytes and regained in zygotes, probably due to the global hyperacetylation at gene dense regions in zygotes. The H3K27ac disappeared at the TSSs of these maternal genes since early 2‐cell. The Polycomb group (PcG) target genes as controls showed no H3K27ac enrichment during MZT (Fig 1F). For the retrotransposon ERVLs, which is one group of the highly expressed repeat elements during MZT, had higher H3K27ac levels at their TSSs than their surrounding regions (Fig EV1G). These results revealed that H3K27ac may prime major ZGA genes and ERVL retrotransposons for their activation during ZGA.

### H3K27ac correlates with H3K4me3 and open chromatin

**Figure 2:**
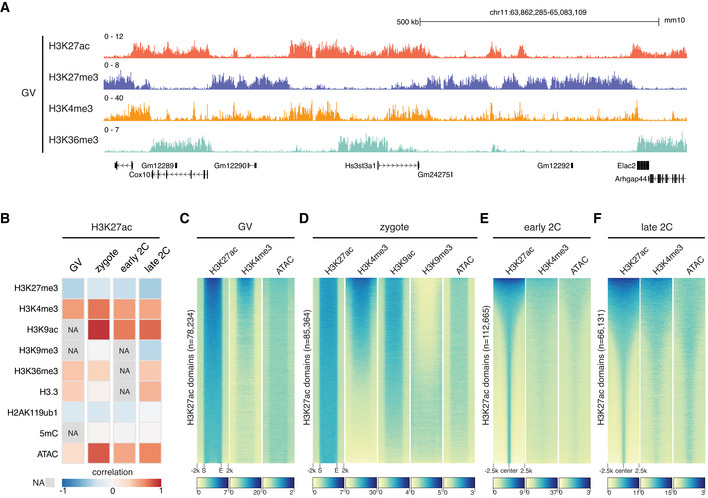
Relationship of H3K27ac with other epigenetic markers AGenome browser view of H3K27ac and other histone modifications signals in GV oocytes.BGlobal spearman correlations of H3K27ac with other epigenetic markers at different stages. NA—data not available. Bin size: 5 kb.C, DHeatmap showing other epigenetic marker signals at H3K27ac domains in GV oocytes (C) and zygotes (D). S—domain start position; E—domain end position.E, FHeatmap showing the enrichment of H3K4me3 and open chromatin (ATAC) around H3K27ac domain centers in early 2‐cell (E) and late 2‐cell embryos (F).

The noncanonical broad H3K27ac distributions (Fig 1) are reminiscent of the noncanonical broad H3K4me3 domains previously reported in mouse oocytes and zygotes (Dahl *et al*, 2016; Zhang *et al*, 2016). This led us to investigate the relationship between H3K27ac and H3K4me3 and other epigenetic markers during MZT. We found that the broad H3K27ac domains were mutually exclusive to H3K27me3 in GV oocytes (Fig 2A), and they were negatively correlated at all the stages as expected (Fig 2B). The H3K27ac showed similar but not identical distributions to H3K4me3 and these two histone modifications were strongly correlated in GV oocytes and zygotes (Fig 2A and B). Detailed examination of the broad domains of H3K27ac and H3K4me3 suggested that they shared the same boundaries in GV oocytes (Fig 2C) and zygotes (Fig 2D). In early 2‐cell and late 2‐cell embryos, as most of the H3K27ac domains were resolved to narrow peak patterns, H3K27ac exhibited reduced but still high correlation with H3K4me3 (Fig 2E and F). The H3K27ac also exhibited high correlation with open chromatin measured by ATAC‐seq (Wu *et al*, 2016) at all the stages analyzed (Fig 2B–F), with the highest correlation in zygotes. The broad H3K27ac domains in zygotes colocalized with open chromatin and shared the same boundaries (Fig 2D).

In addition to H3K27ac, we analyzed H3K9ac distributions in zygotes and 2‐cell embryos (Yang *et al*, 2021; Fig 2B). H3K9ac exhibited a similar distribution with H3K27ac in zygotes (Figs 2D and EV1H), suggesting hyperacetylation of multiple sites on histones in zygotes (Wang *et al*, 2007). However, the reprogramming dynamics of H3K27ac and H3K9ac were distinct (Fig EV1H). While H3K27ac was reprogrammed from zygotes to early 2‐cell embryos, H3K9ac remained largely unchanged from zygote to early 2‐cell and late 2‐cell stages. This observation is consistent with the fact that different acetyltransferases are responsible for depositing these two histone modifications (Shvedunova & Akhtar, 2022). Furthermore, we found that H3K27ac exhibited positive correlation with H3K36me3 (Xu *et al*, 2019) and H3.3 (Ishiuchi *et al*, 2020) at all the stages analyzed (Fig 2B).

### Allelic reprogramming of H3K27ac in mouse early embryos

**Figure 3:**
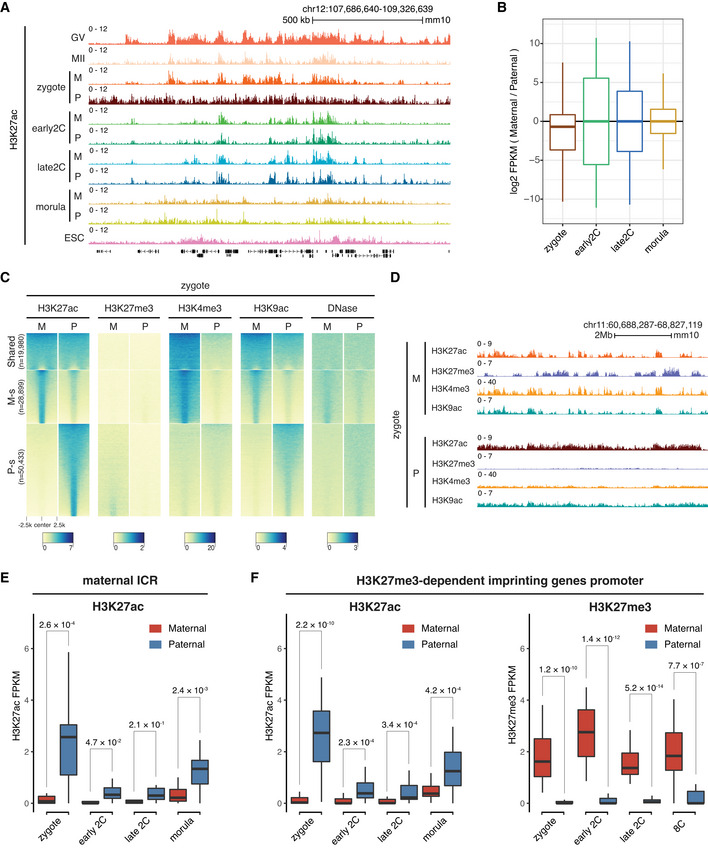
Allelic reprogramming of H3K27ac in mouse early embryos Genome browser snapshot showing the allele‐specific landscape of H3K27ac in early embryos. M—maternal allele; P—paternal allele.Boxplot showing the allelic bias of H3K27ac between maternal allele and paternal allele at different stages. Bin size: 1 kb.Allele‐specific distributions of H3K27ac and other epigenetic markers in zygotes. Each row is a zygotic H3K27ac domain. The H3K27ac domains were classified into three groups using k‐means clustering: Shared—common H3K27ac domains for both maternal and paternal alleles in zygotes; M‐s—maternal allele‐specific H3K27ac domains; P‐s—paternal allele‐specific H3K27ac domains.Genome browser view showing the allelic landscape of H3K27ac and other histone modifications in zygotes.Comparison of H3K27ac signals at maternal imprinting control regions (ICR, *n* = 17) between the maternal allele and paternal allele at different stages. The *P*‐values were derived from one‐sided *t*‐test. The list of maternal ICRs were retrieved from (Xie *et al*, 2012).Comparison of H3K27ac signals at H3K27me3‐dependent imprinted gene promoters (*n* = 27, ± 2 kb of TSS) between the maternal allele and paternal allele at different stages. The *P*‐values were derived from one‐sided *t*‐test. The list of H3K27me3 imprinted genes were from Liu *et al* (2020). Data information: For boxplots in (B), (E), and (F), the central band represents the median. The lower and upper edges of the box represent the first and third quartiles, respectively. The whiskers of the boxplot extend to 1.5 times interquartile range (IQR).

**Figure EV2:**
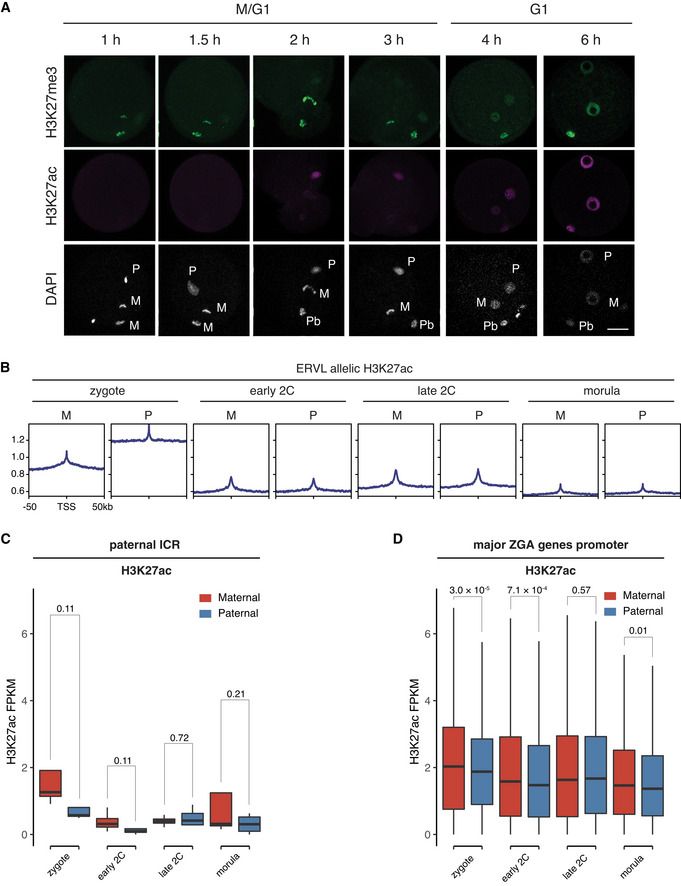
The allele‐specific level of H3K27ac Immunostaining images of fertilized 1‐cell embryos stained with anti‐H3K27me3 and anti‐H3K27ac at indicated time points post fertilization. M—maternal DNA; P—paternal DNA; Pb—polar body. Scale bar: 20 μm.Allele‐specific enrichment of H3K27ac signals at ERVL retrotransposons at different stages. M—maternal allele; P—paternal allele.Comparison of H3K27ac signals at paternal imprinting control regions (ICR, *n* = 4) between the maternal allele and paternal allele at different stages. The *P*‐values were derived from one‐sided *t*‐test. The list of paternal ICRs were retrieved from (Xie *et al*, 2012).Comparison of H3K27ac signals at major ZGA genes promoters (*n* = 2,773, ± 2 kb of TSS) between the maternal allele and paternal allele at different stages. The *P*‐values were derived from two‐sided *t*‐test. Data information: For boxplots in (C) and (D), the central band represents the median. The lower and upper edges of the box represent the first and third quartiles, respectively. The whiskers of the boxplot extend to 1.5 times interquartile range (IQR).

Due to their initial differences, the maternal allele and paternal allele usually have different epigenomic reprogramming dynamics after fertilization. Time‐course immunostaining of H3K27ac after fertilization indicated that the paternal allele established the H3K27ac *de novo* as early as 2 hours post *in vitro* fertilization (hpi), while the maternal allele began to gain H3K27ac from 4 hpi (Fig EV2A). Since two distantly related strains B6D2F1/J (maternal) and PWK/PhJ (paternal) were used to generate embryos for the H3K27ac profiling, we were able to delineate the allele‐specific landscapes of H3K27ac in early embryos (Fig 3A). In zygotes, the maternal allele and paternal allele had different acetylation patterns, with the paternal allele exhibited even larger broad domains and higher acetylation level than the maternal allele (Fig 3A and B). The H3K27ac of two alleles became globally equalized rapidly after the first cleavage (Fig 3B), demonstrating a quick reprogramming of H3K27ac.

As the maternal and paternal alleles exhibited different acetylation patterns mainly in zygotes, we grouped all the H3K27ac domains in zygotes into three clusters: shared domains between the two alleles, maternal‐specific and paternal‐specific domains, and investigated the relationships of these domains with other allelic epigenetic markers (Fig 3C and D). We found that H3K27ac inversely correlated with H3K27me3 on both alleles (Fig 3C). For the paternal‐specific H3K27ac domains, the maternal allele showed H3K27me3 signals while the paternal allele had almost no H3K27me3 (Fig 3C and D), which suggests H3K27me3 may be a barrier for *de novo* H3K27 acetylation in zygotes (Zenk *et al*, 2017). The H3K27ac exhibited high correlation with H3K4me3 on the maternal, but not the paternal allele (Fig 3C and D), which could be due to the overall low level of H3K4me3 on the paternal allele in zygotes. The H3K9ac exhibited a highly similar pattern to H3K27ac for both maternal and paternal alleles (Fig 3C and D). The chromatin openness measured by DNase‐seq (Lu *et al*, 2016) also exhibited a pattern similar to that of H3K27ac for both maternal and paternal alleles in zygotes (Fig 3C and D). Allelic H3K27ac enrichment analyses for ERVL retrotransposons showed that ERVLs had a higher level of H3K27ac on the paternal allele than the maternal allele (Fig EV2B), which is consistent with the global higher H3K27ac level of the paternal allele in zygotes. Starting from early 2‐cell, the H3K27ac enrichment at ERVLs had no difference between the two alleles (Fig EV2B).

The parental‐origin dependent expression of imprinted genes is controlled by either DNA methylation‐dependent imprinting (Bartolomei & Ferguson‐Smith, 2011) or H3K27me3‐dependent noncanonical imprinting (Inoue *et al*, 2017; Chen & Zhang, 2020). To explore a potential relationship between H3K27ac and genomic imprinting, we investigated H3K27ac enrichment at the imprinting control regions (ICRs; Xie *et al*, 2012) and the H3K27me3‐dependent imprinted genes. For maternal ICRs, the H3K27ac level was higher on the paternal allele compared to that on the maternal allele (Fig 3E), even at stages such as zygote and early 2‐cell embryos when most of these genes are not expressed. By contrast, for paternal ICRs, the trend was that the maternal allele showed higher H3K27ac levels than that of the paternal allele at zygote and early 2‐cell stages (Fig EV2C). The nonsignificant *P*‐value could be due to that there are only 4 paternal ICRs in mice. For H3K27me3‐dependent imprinted genes, the H3K27ac level was also significantly higher on the paternal allele than that of the maternal allele for all the embryo stages analyzed (Fig 3F). As controls, the major ZGA genes promoters showed largely comparable levels of H3K27ac in both alleles (Fig EV2D). These results suggest that for both DNA methylation‐dependent ICRs and H3K27me3‐dependent imprinted genes, the active alleles are premarked with H3K27ac even before their expression during embryonic development.

### CBP/p300 inhibition in zygotes leads to H3K27ac loss, ZGA failure, and developmental arrest

**Figure 4:**
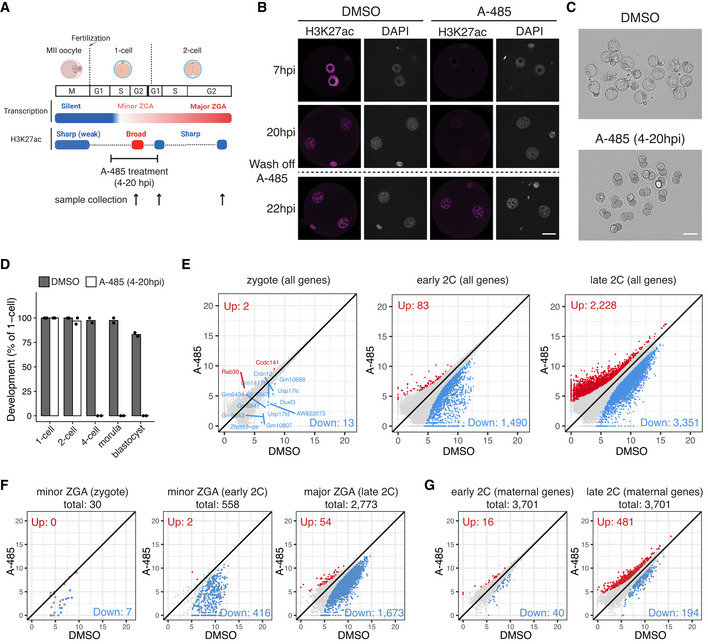
CBP/p300 inhibition in zygotes leads to H3K27ac loss, ZGA failure, and development arrest Schematic illustration of transient CBP/p300 inhibition from zygote to early 2‐cell with A‐485. The cell cycle stages were based on (Abe *et al*, 2018). The arrows indicate the time points of sample collection for RNA‐seq.Immunostaining of H3K27ac in A‐485‐treated embryos versus control (DMSO‐treated) at different time points. Scale bar: 20 μm.Images of embryos treated with DMSO (control) and A‐485 at 96 hpi. Scale bar: 80 μm.Developmental rate of embryos treated with A‐485 versus control.Scatter plots showing the whole transcriptome changes in zygotes, early 2‐cell, and late 2‐cell embryos after CBP/p300 inhibition.Scatter plots showing the expression level changes of zygote minor ZGA genes (*n* = 30), early 2‐cell minor ZGA genes (*n* = 558), and major ZGA genes (*n* = 2,773) after CBP/p300 inhibition.Scatter plot showing the expression level changes of maternal decay genes (*n* = 3,701) in early 2‐cell and late 2‐cell embryos after CBP/p300 inhibition.

**Figure EV3:**
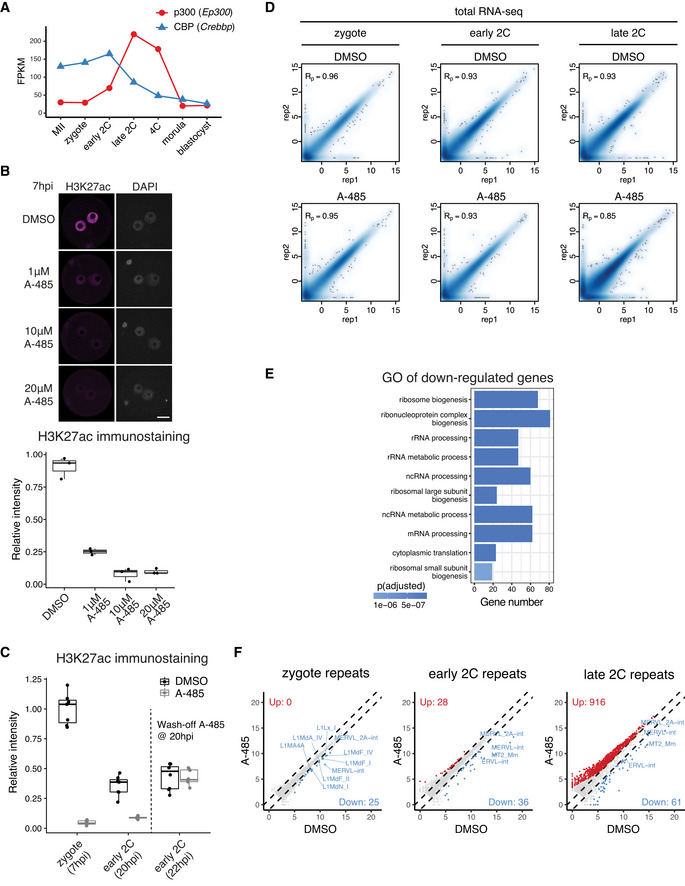
Transcriptome comparison of CBP/p300 inhibition versus control Expression level dynamics of *Ep300* (p300) and *Crebbp* (CBP) in MII oocyte and preimplantation embryos.Determination of optimal A‐485 concentration to inhibit CBP/p300 by H3K27ac immunostaining. The embryos (3 biological replicates in each condition) were treated with A‐485 starting at 4 hpi, and images were taken at 7 hpi. Scale bar: 20 μm.Immunostaining quantification of H3K27ac relative intensities in Fig 4B (6–12 biological replicates in each condition).Pearson correlation of RNA‐seq replicates at zygote, early 2‐cell, and late 2‐cell stage for control (DMSO‐treated) and CBP/p300 inhibition (A‐485‐treated).Gene Ontology (GO) enrichment for the down‐regulated genes at early 2‐cell stage after A‐485 treatment.Scatter plot showing the expression level changes of repeat elements after CBP/p300 inhibition. Data information: For boxplots in (B) and (C), the central band represents the median. The lower and upper edges of the box represent the first and third quartiles, respectively. The whiskers of the boxplot extend to 1.5 times interquartile range (IQR).

Our H3K27ac profiling revealed the unexpected noncanonical broad acetylation domains in zygotes. We next attempted to explore the functional implications of such broad acetylation in zygotes. Previous studies have shown that H3K27ac is deposited by the two paralogue acetyltransferases CBP/p300 (Tie *et al*, 2009). Analysis of the CBP/p300 expression indicated that they were highly expressed and dynamically regulated in MII oocytes and preimplantation embryos (Fig EV3A). To examine the functional consequence of removing acetylation in zygotes, we used a highly selective inhibitor A‐485 (Lasko *et al*, 2017) to inhibit the catalytic activity of CBP/p300 from 4 to 20 hpi (Fig 4A), without affecting CBP/p300 binding (Narita *et al*, 2021). We determined the optimal concentration of A‐485 treatment at 10 μM (Fig EV3B), which completely abolished H3K27ac at 7 hpi (3 h treatment), demonstrating that CBP/p300 is responsible for depositing H3K27ac in zygotes. The CBP/p300 inhibition was reversible as global H3K27ac was restored to the control level after washing off A‐485 (Figs 4B and EV3C). The transient inhibition of CBP/p300 from zygote to early 2‐cell led to developmental arrest at 2‐cell stage for all the embryos (Fig 4C and D), demonstrating that the CBP/p300 acetyltransferase activity in zygotes is critical for mouse early preimplantation development.

To determine how CBP/p300 inhibition in zygote causes such a severe embryonic developmental arrest, we performed total RNA‐seq of zygotes, early 2‐cell and late 2‐cell embryos with (A‐485‐treated) or without (DMSO‐treated) the inhibitor treatment (Fig EV3D) and investigated whether ZGA genes were affected. There are two waves of ZGA in mouse embryos—minor ZGA occurring from late 1‐cell to early 2‐cell stage and major ZGA occurring at late 2‐cell stage (Schulz & Harrison, 2019). To dissect the fine‐scale impact of CBP/p300 inhibition, we further separated minor ZGA genes into zygote minor ZGA genes (*n* = 30, Dataset EV1) and early 2‐cell minor ZGA genes (*n* = 558, Dataset EV1, see Materials and Methods). Comparative whole transcriptome analysis (Fig 4E) showed the down‐regulation of 13 genes in zygotes (Dataset EV2), 1,490 genes in early 2‐cell embryos (Dataset EV3), and 3,351 genes in late 2‐cell embryos (Dataset EV4). Most of the down‐regulated genes in zygotes were minor ZGA genes including *Duxf3*, *Usp17lc*, and *Usp17ld*, etc., and the functions of the down‐regulated genes at early 2‐cell stage were enriched in ribosome biogenesis and RNA processing, etc. (Fig EV3E), indicating aberrant ZGA. Indeed, 7 of the 30 zygote minor ZGA genes and 416 (74.6%) of the 558 early 2‐cell minor ZGA genes were significantly down‐regulated after CBP/p300 inhibition (Fig 4F). Most of the other zygote minor ZGA genes also showed the trend of down‐regulation, but their *P*‐values were not significant, probably due to their very low expression levels (Fig 4F). By contrast, maternal RNA decay at early 2‐cell stage was not affected (Fig 4G). These results suggested a direct effect of CBP/p300 on minor ZGA. At late 2‐cell stage, many more genes were down‐regulated or up‐regulated (Fig 4E) for ZGA genes (Fig 4F) and maternal RNAs (Fig 4G). In addition, several types of repeat elements including the LINE1, MERVL, and MT2, which are highly expressed retrotransposons during ZGA, were also down‐regulated in zygotes, early 2‐cell, and late 2‐cell embryos after CBP/p300 inhibition (Fig EV3F). These results demonstrate that the CBP/p300 acetyltransferase activities are required for minor ZGA, major ZGA, and early preimplantation development.

### CBP/p300 inhibition affects putative enhancers at early 2‐cell

**Figure 5:**
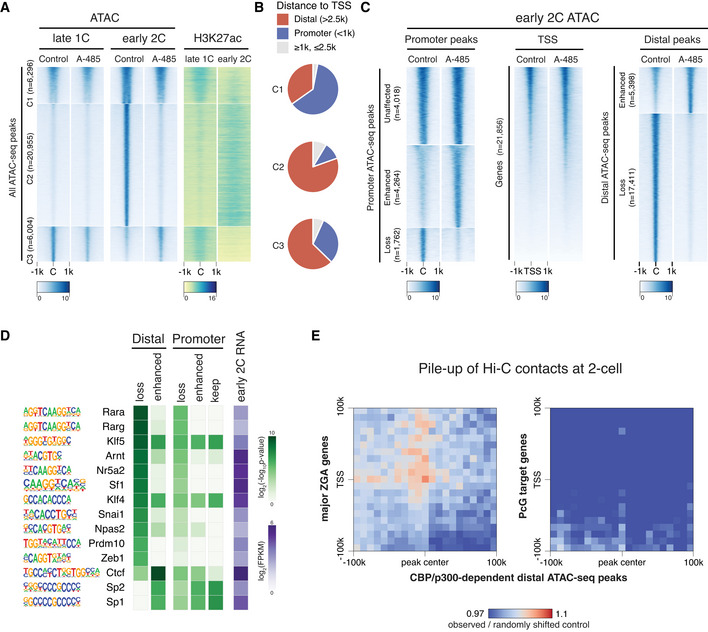
CBP/p300 inhibition affects chromatin opening of putative enhancers at early 2‐cell stage Heatmap showing ATAC‐seq signals at late 1‐cell and early 2‐cell under control and A‐485‐treated conditions, and the corresponding H3K27ac signals at these ATAC‐seq peaks. All the ATAC‐seq peaks were classified into three groups based on the changes from late 1‐cell to early 2‐cell in control condition. C1—keep open from late 1‐cell to early 2‐cell; C2—newly opened at early 2‐cell; C3—reduced opening at early 2‐cell.Ratio of distal ATAC‐seq peaks (>2.5 kb to TSS) and promoter ATAC‐seq peaks (<1 kb to TSS) in group C1‐3 of panel A.Heatmap showing the impact of CBP/p300 inhibition on chromatin openness of different regions (promoter, TSS and distal regions) at early 2‐cell stage.Transcription factor (TF) motif enrichment at distal and promoter ATAC‐seq peaks with different responses to CBP/p300 inhibition. Only TFs with expression level of FPKM≥5 at early 2‐cell stage were considered.Pile‐up of Hi‐C contacts at 2‐cell stage between CBP/p300‐dependent distal ATAC‐seq peaks (x‐axis) and major ZGA genes TSS regions (y‐axis, left panel). The same analysis was also performed between CBP/p300‐dependent distal ATAC‐seq peaks and PcG target genes TSS (right panel) as a control.

**Figure EV4:**
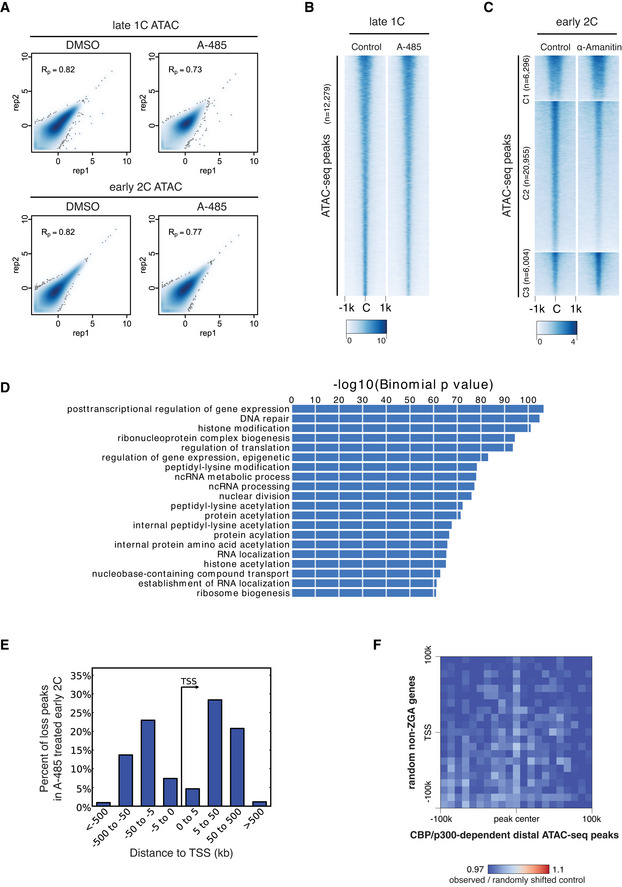
Chromatin accessibility comparison of CBP/p300 inhibition versus control Pearson correlation of ATAC‐seq replicates for DMSO‐treated and A‐485‐treated late 1‐cell and early 2‐cell.ATAC‐seq signal comparison for control and A‐485‐treated late 1‐cell. C—peak center.ATAC‐seq signal comparison for control and minor ZGA inhibition (treated with α‐Amanitin) at early 2‐cell stage. The ATAC‐seq peaks were the same as in Fig 5A. The ATAC‐seq data of control early 2‐cell and α‐Amanitin‐treated early 2‐cell were from GEO with accession GSM1933921, GSM1933922, GSM1933923, and GSM2108702 (Wu *et al*, 2016).Gene Ontology (GO) enrichment for the nearest genes of the ATAC‐seq peaks that were lost under A‐485 treatment in early 2‐cell, using GREAT analysis (McLean *et al*, 2010).Distance to TSS for the ATAC‐seq peaks that were lost after A‐485 treatment in early 2‐cell embryos.Pile‐up of Hi‐C contacts at 2‐cell stage between CBP/p300‐dependent distal ATAC‐seq peaks (x‐axis) and randomly selected non‐ZGA genes TSS regions (*n* = 2,773, matched chromosome distribution with major ZGA genes).

Next, we asked how the inhibition of the CBP/p300 acetyltransferase activity in zygotes led to the down‐regulation of major ZGA genes. One possibility is that acetylation by CBP/p300 would open up chromatin before major ZGA. To test this possibility, we performed ATAC‐seq for late 1‐cell and early 2‐cell embryos with CBP/p300 inhibition (A‐485‐treated) and control (DMSO‐treated) conditions (Fig EV4A). Results indicated that the chromatin accessibility at late 1‐cell was largely unaffected by CBP/p300 inhibition (Figs 5A and EV4B). By contrast, the newly opened regions in control early 2‐cell (Fig 5A, cluster C2), coincide with the gain of H3K27ac at early 2‐cell stage, remained closed after CBP/p300 inhibition by A‐485 (Fig 5A). Since CBP/p300 activity is required for the activation of many minor ZGA genes, loss of chromatin accessibility after CBP/p300 inhibition can be either an effect of minor ZGA defects or a direct effect of CBP/p300 inhibition. To clarify this, we analyzed the ATAC‐seq data of early 2‐cell after minor ZGA inhibition by treating with α‐Amanitin (Wu *et al*, 2016). Results showed that α‐Amanitin treatment only had a milder effect on the ATAC‐seq peaks at early 2‐cell (Fig EV4C), indicating the chromatin opening at early 2‐cell mainly depends on CBP/p300 activity.

Gene ontology (GO) enrichment analysis (McLean *et al*, 2010) showed that the nearest genes to those ATAC‐seq peaks that were lost after CBP/p300 inhibition (CBP/p300‐dependent ATAC‐seq peaks) at early 2‐cell stage were enriched for posttranscriptional regulation of gene expression, histone modification and acetylation, etc. (Fig EV4D). For the CBP/p300‐dependent ATAC‐seq peaks, most of them were distal peaks far away from TSS (Figs 5B and EV4E). To further confirm this observation, we classified all ATAC‐seq peaks into promoter or distal peaks and investigated their response to A‐485 treatment. Results showed that most distal peaks (*n* = 17,411, 76.3%) were lost after A‐485 treatment (Fig 5C). However, most promoter peaks showed unaffected or enhanced signals than loss (8,282, 82.5% vs. 1,762, 17.5%) upon CBP/p300 inhibition (Fig 5C). These results demonstrate that transient inhibition of CBP/p300 acetyltransferase activity mainly affects distal chromatin opening at early 2‐cell stage.

The distal and promoter open chromatin at early 2‐cell stage showed different responses to CBP/p300 inhibition (Fig 5A–C). Given that CBP/p300 does not recognize specific DNA sequence motifs (Calo & Wysocka, 2013), we hypothesized that different transcription factors (TFs) that recruit CBP/p300 may bind to proximal or distal elements, which may cause their differential responses to CBP/p300 inhibition. To examine this possibility, TFs expressed at early 2‐cell with FPKM at least 5 were included in the motif enrichment analysis. Results indicated that motifs of the retinoic acid receptors (RARs), Nr5a2, Sf1, Prdm10, and Zeb1 were specifically enriched in CBP/p300‐dependent (lost after CBP/p300 inhibition) open chromatins, while Sp1 and Sp2 motifs were enriched in CBP/p300‐independent open chromatins (Fig 5D). Notably, among these TFs, RARs and Nr5a2 have been previously implicated in regulating 2‐cell stage programs and preimplantation development (Wu *et al*, 2016; Iturbide *et al*, 2021; preprint: Gassler *et al*, 2022).

Next, we investigated whether CBP/p300‐dependent distal open chromatins at 2‐cell stage may regulate ZGA by directly contacting the major ZGA genes. To this end, we analyzed the Hi‐C data in mouse 2‐cell embryos (Du *et al*, 2017) and performed the pile‐up analysis between the CBP/p300‐dependent distal open chromatin regions and the major ZGA genes. We found that the CBP/p300‐dependent distal open regions showed higher contacts with the major ZGA genes than random controls (Fig 5E). By contrast, these distal open regions showed no higher than random contacts with the PcG target genes (Fig 5E) or randomly selected non‐ZGA genes (Fig EV4F). These results indicate that the CBP/p300‐dependent distal open chromatin regions at early 2‐cell stage may function as enhancers of major ZGA genes.

### HDAC activity prevents premature expression of developmental genes and aberrant ZGA

**Figure 6:**
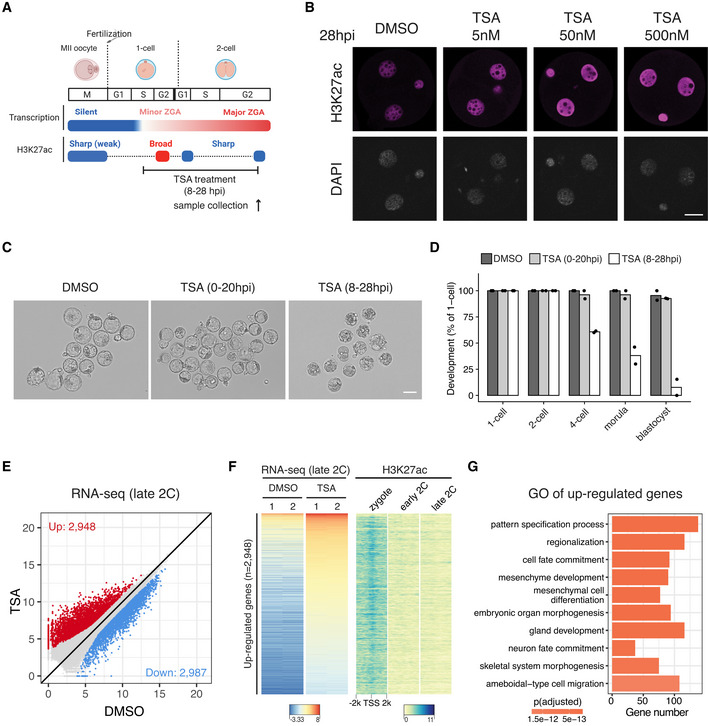
HDAC inhibition blocks deacetylation from zygote to 2‐cell embryos and leads to premature expression of developmental genes Schematic illustration of HDAC inhibition from zygotes to late 2‐cell embryos with TSA (Trichostatin A). The cell cycle stages were based on (Abe *et al*, 2018). The arrow indicates the time point of sample collection for RNA‐seq.Immunostaining of H3K27ac in TSA‐treated embryos under different concentrations versus control (DMSO‐treated) at 28 hpi. Scale bar: 20 μm.Images of embryos treated with DMSO (control) or TSA at 96 hpi. Scale bar: 80 μm.Developmental rate of embryos treated with TSA at different time points (0–20 hpi and 8–28 hpi) versus control.Late 2‐cell embryo whole transcriptome comparison between HDAC inhibition (TSA‐treated) and control (DMSO‐treated).Heatmap showing the up‐regulated genes after TSA treatment at late 2‐cell stage and the corresponding H3K27ac dynamics at the promoter of these up‐regulated genes.Gene Ontology (GO) enrichment analysis of the up‐regulated genes after HDAC inhibition.

**Figure EV5:**
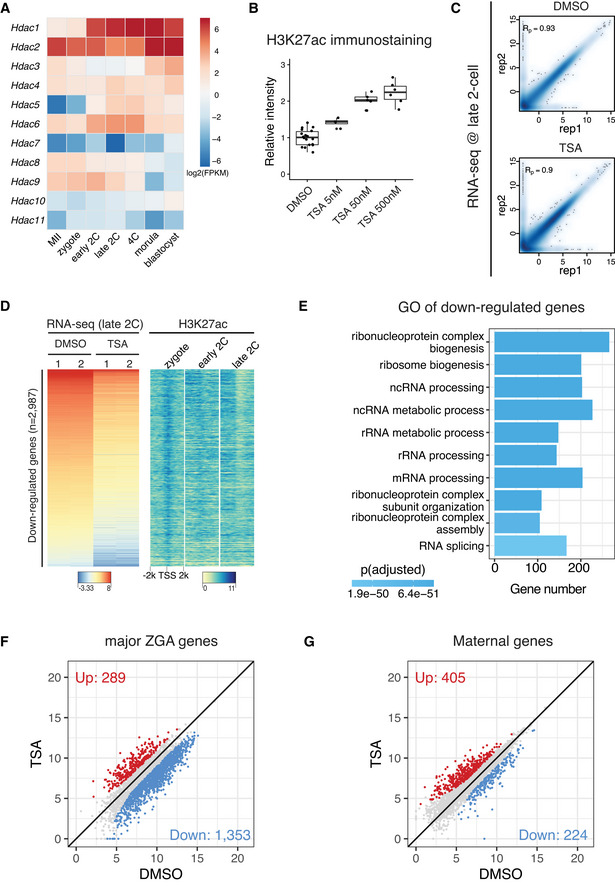
Transcriptome comparison of HDAC inhibition versus control Expression dynamics of HDAC genes in mouse MII oocyte and preimplantation embryos.Immunostaining quantification of H3K27ac relative intensities in Fig 6B (4–16 biological replicates in each condition).Pearson correlation of RNA‐seq replicates at late 2‐cell stage for control (DMSO‐treated) and HDAC inhibition (TSA‐treated).Heatmap showing the down‐regulated genes after TSA treatment in late 2‐cell embryos and the corresponding H3K27ac dynamics at the promoters of these down‐regulated genes.Gene Ontology (GO) enrichment analysis of the down‐regulated genes after HDAC inhibition.Scatter plot showing the impact of HDAC inhibition on expression levels of major ZGA genes.Scatter plot showing the impact of HDAC inhibition on expression levels of maternal decay genes. Data information: For boxplots in (B), the central band represents the median. The lower and upper edges of the box represent the first and third quartiles, respectively. The whiskers of the boxplot extend to 1.5 times interquartile range (IQR).

We next asked about the functional implications of the rapid transition from the broad acetylation domains in zygotes to canonical domains in early 2‐cell embryos (Fig 1B). Removal of the broad acetylation domains from zygote to early 2‐cell would require HDACs, and several HDACs were highly expressed in early 2‐cell embryos (Fig EV5A). To reveal the function of HDACs activity in this context, we used the specific and potent HDAC inhibitor Trichostatin A (TSA) to treat the 1‐cell to 2‐cell embryos from 8 to 28 h post *in vitro* fertilization (Fig 6A). Since TSA treatment at 50 nM caused hyperacetylation to a comparable level as that of 500 nM (Figs 6B and EV5B), we used 50 nM for subsequent experiments. We found that transient HDAC inhibition (8–28 hpi) caused embryo developmental arrest (Fig 6C and D). Given that HDAC inhibition from 0 to 20 h post fertilization did not cause embryo developmental arrest (Fig 6C and D), the inhibitor likely affects an event that takes place between 20–28 h post fertilization, which coincides with zygote to 2‐cell transition.

To investigate how inhibition of HDAC activity during zygote to 2‐cell transition causes embryo developmental arrest, we performed total RNA‐seq of late 2‐cell embryos in HDAC inhibition (TSA‐treated 8–28 hpi) and control (DMSO‐treated 8–28 hpi) conditions (Fig EV5C). Comparative transcriptome analysis indicated that 2,948 genes were up‐regulated and 2,987 genes were down‐regulated after HDAC inhibition (Fig 6E, Dataset EV5). The up‐regulated genes coincided with deacetylation around their TSSs from zygote to 2‐cell (Fig 6F), indicating that they are likely direct targets of HDACs. By contrast, the TSSs of down‐regulated genes showed high levels of acetylation at both zygote and 2‐cell stages (Fig EV5D), indicating that their down‐regulation is likely due to secondary effects. Detailed examination of the up‐regulated genes revealed most of them were developmental genes that should be expressed at later developmental stages (Fig 6G). On the other hand, most of the down‐regulated genes were major ZGA genes, and a subset of up‐regulated genes were maternal genes (Fig EV5D–G), indicating that the premature expression of developmental genes may disrupt both ZGA and maternal decay. These results demonstrate that the HDACs activities, which mediated the broad‐to‐canonical acetylation transition from zygote to 2‐cell, have a critical role in preventing premature expression of developmental genes and in ensuring normal ZGA gene expression.

## Discussion

By profiling the well‐recognized active chromatin marker H3K27ac in mouse oocytes and preimplantation embryos, here we report the rapid turn‐over of H3K27ac during MZT (Fig 1). The H3K27ac undergoes three successive rounds of global transitions during MZT. The GV oocytes show noncanonical large broad H3K27ac domains, which are mutually exclusive to H3K27me3 for both gene dense regions and gene deserts. Such broad acetylation domains are removed in MII oocytes (first wave of global transition), with a few narrow domains remain. This observation is consistent with a previous report that HDACs can associate with chromatin during meiosis (Kim *et al*, 2003). Intriguingly, mouse zygotes regain the large broad H3K27ac domains shortly after fertilization (second wave of global transition), mainly in gene dense regions. In addition to H3K27ac, H3K9ac also displays highly similar broad domains in zygotes, suggesting a global hyperacetylation of multiple histone sites in mouse zygotes (Wang *et al*, 2007). Such broad acetylation domains indicate that H3K27ac cannot serve as a marker for enhancers in oocytes and zygotes as in somatic cells. However, the broad acetylation domains may contribute to the establishment of a permissive state for transcription in late zygotes (Schultz, 2002). After the first cell cleavage, the broad H3K27ac is quickly reprogrammed to a canonical pattern in early 2‐cell embryos (third wave of global transition). The H3K27ac distributions in early 2‐cell (before major ZGA) and late 2‐cell embryos (major ZGA) exhibit a similar pattern, indicating a preconfiguration of H3K27ac before ZGA. Indeed, we observed that H3K27ac marks genomic regions that are primed for activation of the major ZGA genes, retrotransposons, and the active allele of imprinted genes. The ZGA genes in zebrafish and *Drosophila* are also premarked by H3K27ac (Zhang *et al*, 2018) and H4K16ac (Samata *et al*, 2020), respectively, suggesting that the preconfiguration of histone acetylation for gene activation is an evolutionarily conserved process.

We provided evidence that the broad H3K27ac domains in zygote are deposited by CBP/p300 (Fig 4). We further demonstrated that the catalytic activity of CBP/p300 is critical for minor ZGA, and for opening the distal chromatin regions at early 2‐cell (Figs 4 and 5), both of which are important for major ZGA. The CBP/p300‐dependent distal opening elements may function as enhancers as they make direct contacts with the promoters of major ZGA genes. Unlike the distal regions, the opening of most of the promoter regions is independent of CBP/p300 activities, indicating that promoter opening is regulated by other factors. This observation is different from that in zebrafish. In zebrafish embryos, the opening of both distal and promoter regions is independent of CBP/p300 activity (Miao *et al*, 2022). We identified several transcription factors including RARs, Nr5a2, Sf1, Prdm10, and Zeb1 that may mediate CBP/p300‐dependent distal chromatin opening at mouse early 2‐cell stage (Fig 5D). Future studies using knockout mouse lines should clarify their potential roles. Previous reporter assays indicated that enhancer activity is required for transcription at 2‐cell but not zygotic stage (Schultz, 2002), which is in line with our finding of hyperacetylation at zygote but hypoacetylation at 2‐cell stage. It is possible that the broad H3K27ac domains confer the permissive chromatin state in zygotes. Our results indicate that not only ectopic reporter genes but also endogenous ZGA gene expression in 2‐cell embryos require enhancer activities.

We demonstrated that the timely deacetylation from zygote to 2‐cell (8–28 hpi) by HDACs is critical in preventing premature expression of developmental genes and in ensuring normal ZGA gene expression (Fig 6). This is consistent with the earlier observation that *Hdac1* knock‐down causes elevated expression of a subset of transcripts in late 2‐cell embryos (Ma & Schultz, 2008). Given that multiple HDACs are expressed in preimplantation embryos and they may compensate each other, our work here provides the definite evidence that HDAC activities establish a transcriptionally repressive state in 2‐cell embryos that is important for early development. Among these HDAC genes, *Hdac1* and *Hdac2* are highly expressed in early 2‐cell embryos (Fig EV5A), which coincide with the extensive deacetylation at early 2‐cell stage. This indicates that HDAC1/2 may be the key factor functioning in the process, which is supported by the recent demonstration that HDAC1/2 is required to safeguard ZGA in mice (Dang *et al*, 2022). It should be noted that the essential role of HDACs activities during MZT reported here is not in conflict with the previous work showing that HDAC inhibition (HDACi) improves the efficiency of somatic cell nuclear transfer (SCNT; Kishigami *et al*, 2006; Rybouchkin *et al*, 2006; Matoba *et al*, 2014; Yang *et al*, 2021). In SCNT, reconstructed embryos are treated with HDACi only during the first several hours after egg activation (i.e., 0–10 h), a time window when hyperacetylation is being established (Fig 1). Consistently, we also show that 0–20 hpi TSA treatment does not affect preimplantation development. However, prolonged TSA treatment impedes the broad‐to‐canonical H3K27ac transition before major ZGA and impairs both normal development (Fig 6) and SCNT (Kishigami *et al*, 2006). Thus, the precisely regulated HDACs activity is critical for normal and SCNT embryo development.

Our study has demonstrated that timely CBP/p300 and HDACs activities modulate normal ZGA, which coincides with the dynamic changes of H3K27ac during mouse MZT. However, CBP/p300 and HDACs have relatively low substrate specificities (Weinert *et al*, 2018). They catalyze the acetylation and deacetylation of not only H3K27ac but also other histones lysine residues and nonhistone proteins. Thus, it remains unknown whether the effects of CBP/p300 and HDACs in regulating mouse ZGA is through H3K27ac only or other histone lysine residues/nonhistone substrates are also involved. Recent studies suggest that H3K27ac is not required for enhancer functions in mESCs (Martire *et al*, 2020; Zhang *et al*, 2020; Sankar *et al*, 2022). However, other studies indicated that H3K27ac is required for enhancer activities (Raisner *et al*, 2018). Furthermore, it is not clear whether the observation in mESCs is applicable to mouse embryos. It is possible that the different histone acetylation modifications catalyzed by CBP/p300 are redundant for enhancer activities. Nevertheless, we demonstrate an important role of CBP/p300 acetyltransferase activities in regulating minor ZGA and distal chromatin opening right before mouse major ZGA, and a role of HDACs activities in preventing premature expression of developmental genes, which collectively confer normal ZGA during mouse MZT.

## Materials and Methods

### Collection of mouse oocytes and embryos

All animal experiments were performed in accordance with the guidelines of the Institutional Animal Care and Use Committee at Harvard Medical School. Collection of oocytes and *in vitro* fertilized embryos was described previously (Chen & Zhang, 2019; Chen *et al*, 2021). For allelic H3K27ac CUT&RUN profiling experiments, oocytes were collected from 6‐ to 8‐week‐old B6D2F1/J female mice (Jax 100006), and sperm were collected from 8‐ to 10‐week‐old PWK/PhJ male mice (Jax 003715). For CBP/p300 and HDAC inhibition experiments, both oocytes and sperm were retrieved from 6‐ to 8‐week‐old B6D2F1/J mice. The time when capacitated sperm were added to cumulus‐oocyte complexes in Human Tubal Fluid (HTF, Millipore) was considered as 0 h post *in vitro* fertilization (0 hpi). Embryos were cultured in potassium simplex optimized medium (KSOM, Millipore) at 37°C under 5% CO_2_ with air.

### Transient treatment with A‐485 and TSA

To transiently inhibit CBP/p300, embryos were cultured in KSOM containing 10 μM A‐485 (Tocris) between 4 and 20 hpi. For transient inhibition of HDACs, embryos were cultured in KSOM containing 50 nM TSA (Sigma) between 0–20 or 8–28 hpi. Embryos were washed at least six times when they were transferred between different culture conditions.

### Whole‐mount immunostaining

Immunostaining, image acquisition, and analyses were performed as described previously (Inoue *et al*, 2018). Embryos were fixed with 3.7% paraformaldehyde and 0.2% Triton X‐100 for 20 min at room temperature. After washing 4 times with PBS containing 1% BSA, samples were incubated with the rabbit anti‐H3K27ac antibody (1/500; Millipore, 07–360) and/or the mouse anti‐H3K27me3 antibody (1/500; Active Motif, 61017) as primary antibodies at 4°C overnight. Then, samples were washed with PBS/BSA and incubated with Alexa flour 568 donkey anti‐rabbit IgG and/or Alexa flour 488 donkey anti‐mouse IgG (1/200; Life Technologies) as secondary antibodies for 1 h at room temperature. After washing with PBS/BSA, samples were mounted on a slide containing VectaShield Antifade Mounting Medium with DAPI (Vector Laboratories). Fluorescent intensities were quantified with Zeiss Zen software.

### CUT&RUN library construction

CUT&RUN libraries were generated based on the original protocol (Meers *et al*, 2019) with some modifications to optimize for low‐input cell numbers. For each library, 10 μl BioMag Plus Concanavalin A beads (Polysciences) were washed twice in 1 ml Binding Buffer (20 mM HEPES‐KOH pH7.9, 10 mM KCl, 1 mM CaCl_2_, 1 mM MnCl_2_) and resuspended in 5 μl Binding Buffer. Then, embryos were added to 45 μl Wash Buffer (20 mM HEPES‐NaOH pH7.5, 150 mM NaCl, 0.5 mM Spermidine, 1X Roche Protease Inhibitor Cocktail), mixed with the 5 μl Binding Buffer containing beads, and rotated for 10 min at room temperature. After removing the liquid by putting the tube on a magnet stand, the beads were resuspended in 80 μl antibody Incubation Buffer (1X Wash Buffer with 0.02% Digitonin and 2 mM EDTA) with 1:100 diluted H3K27ac antibody (Millipore, 07–360) and rotated at 4°C overnight. Then, the beads were washed twice with 100 μl Wash Buffer with 0.02% Digitonin. The beads were resuspended in 100 μl Wash Buffer with 0.02% Digitonin and 500 ng/ml pA‐MNase, and rotated for 3 h at 4°C. After washing the beads twice with 200 μl Wash Buffer with 0.02% Digitonin, the beads were resuspended in 200 μl ice‐cold Wash Buffer with 0.02% Digitonin and 4 μl 100 mM CaCl_2_, and incubated at 0°C for 30 min. The reaction was stopped by adding 23 μl 10X Stop Buffer (1,700 mM NaCl, 100 mM EDTA, 20 mM EGTA, 0.02% Digitonin, 250 μg/ml RNase A, 250 μg/ml Glycogen). Then, the tube was incubated at 37°C for 10 min to release the DNA fragments into supernatant. The tube was put on a magnet stand and the supernatant was transferred to a new 1.5 ml LoBind tube (Eppendorf) while the beads were discarded. To elute DNA, 2.5 μl 10% SDS, and 2.5 μl Proteinase K (20 mg/ml) were added to the supernatant and incubated at 55°C for 1 h. Carrier RNA (25 ng, Qiagen) was added to the supernatant before the supernatant was mixed with 200 μl Phenol:Chloroform:Isoamyl Alcohol (Invitrogen) by vortexing. The mixtures were then transferred to a phase‐lock tube (QuantaBio) and centrifuged at 16,000 g for 5 min at room temperature. After transferring the supernatant to a new 1.5 ml LoBind tube, 26 μl 3 M Sodium Acetate, 1 μl Glycogen (20 mg/ml), and 750 μl cold 100% Ethanol were added, mixed by vortexing, and stored at ‐20°C overnight. Then, the tube was centrifuged at 16,000 g for 20 min at 4°C and the supernatant was discarded. The precipitated DNA pellet was washed with 1 ml cold 80% Ethanol, then centrifuged at 16,000 g for 5 min at 4°C, and the supernatant was discarded. Finally, the precipitated DNA pellet was dissolved in 25 μl ultrapure H_2_O. The library was constructed with NEBNext Ultra II DNA Library Prep Kit for Illumina (NEB) following the manufacturer's protocol. Sequencing was performed on the Illumina NextSeq 550 system in paired‐end 75 bp mode.

### RNA‐seq library construction

The total RNA‐seq libraries were generated using the SMART‐Seq Stranded Kit (Takara) following the manufacturer's protocol. Briefly, embryos were suspended in 7 μl RRI water (199 μl ultrapure H_2_O with 1 μl RNase inhibitor). Then, 6 μl shearing master mix (1 μl 10X lysis mix, 1 μl Smart scN6, 4 μl scRT Buffer) was added and incubated at 85°C for 6 min and immediately put on ice for 2 min. For reverse transcription, 7 μl first strand master mix (4.5 μl Smart scTSO mix, 0.5 μl RNase inhibitor, 2 μl SmartScribe RT) was added and incubated at 42°C for 90 min, 70°C for 10 min and held at 4°C. For the first PCR amplification, 28 μl master mix (25 μl 2X SeqAmp Buffer, 1 μl SeqAmp polymerase, 2 μl H_2_O) and 1 μl of each 5′ and 3′ PCR primer were added and incubated at 94°C for 1 min, 10 cycles of 98°C for 15 s, 55°C for 15 s, 68°C for 30 s, 1 cycle of 68°C for 2 min, and held at 4°C. The PCR products were purified with 35 μl SPRI beads (Beckman Coulter), washed twice with 50 μl 80% Ethanol, and eluted in 50 μl H_2_O. The PCR products were purified again with 40 μl SPRI beads, washed twice with 200 μl 80% Ethanol, and eluted in 20 μl scZapR master mix (16.8 μl H_2_O, 2.2 μl 10X ZapR buffer, 1.5 μl scZapR and 1.5 μl preheated and chilled scR‐probes) for 5 min to remove ribosome RNA. Then, the supernatant was transferred into a new PCR tube and incubated at 37°C for 60 min, 72°C for 10 min, and held at 4°C. For second PCR amplification, 80 μl master mix (26 μl H_2_O, 50 μl SeqAmp CB Buffer, 2 μl PCR2 primers, 2 μl SeqAmp polymerase) were added to the 20 μl sample and split into two PCR tubes for amplification at 94°C for 1 min, 10 cycles of 98°C for 15 s, 55°C for 15 s, 68°C for 30 s, 1 cycle of 68°C for 2 min and held at 4°C. The PCR products were purified with 1:1 SPRI beads and eluted in 12 μl H_2_O. The libraries were sequenced on the Illumina NextSeq 550 system in paired‐end 75 bp mode.

### ATAC‐seq library construction

To generate ATAC‐seq libraries, embryos were suspended in 10 μl tagmentation buffer (33 mM Tris–Acetate, 66 mM K‐Acetate, 10 mM Mg‐Acetate, 16% Dimethylformamide, and 0.02% Digitonin) with 0.5 μl Tn5/Adapter complex (Diagenode) and incubated at 37°C for 20 min. The tagmentation was stopped by adding 10 μl stop buffer (100 mM Tris–pH8.0, 100 mM NaCl, 0.4% SDS, 40 μg/ml Proteinase K) then incubated at 55°C for 3 h to release the tagmentation fragments. SDS was quenched by adding 5 μl 25% Tween‐20 and incubated on ice for 10 min. For library PCR amplification, the 25 μl samples were mixed with 30 μl NEBNext High‐Fidelity 2X PCR Master Mix (NEB), 2.5 μl P5 primer (10 μM) and 2.5 μl P7 primer (10 μM). Then, the PCR was performed as 5 min at 72°C, 5 min at 98°C, 16 cycles of 20 s at 98°C, 30 s at 63°C, 60 s at 72°C, and 1 cycle of 5 min at 72°C, held at 4°C. The PCR products were purified with 1:1.6 SPRI beads (96 μl beads), washed twice with 200 μl 80% Ethanol, and eluted in 20 μl H_2_O. The libraries were sequenced on the Illumina NextSeq 550 system in paired‐end 75 bp mode.

### CUT&RUN data analysis

For CUT&RUN sequencing data, the raw paired‐end reads were trimmed with Trimmomatic (v0.39; Bolger *et al*, 2014) to remove adaptors and the trimmed reads with length of at least 35 bp were retained. The cleaned reads were mapped to GRCm38 reference genome using bowtie2 (v2.4.2; Langmead & Salzberg, 2012) with parameters: ‐‐local ‐‐very‐sensitive‐local ‐‐no‐unal ‐‐no‐mixed ‐‐no‐discordant ‐‐dovetail ‐I 10 ‐X 700 ‐‐soft‐clipped‐unmapped‐tlen. PCR duplicates were removed with Picard MarkDuplicates (v2.23.4). The mapped reads were further filtered to retain proper paired reads with fragment length of at least 140 and mapping quality of at least 30. The signal tracks were generated by bamCoverage in deeptools (v3.5.1; Ramirez *et al*, 2016) with 100 bp bin size and normalized with FPKM.

Since the H3K27ac showed broad or narrow patterns at different stages of early embryos, the FPKM values were not directly comparable among stages. To make the FPKM values comparable, we followed the scaling method previously used for characterizing broad H3K4me3 domains in mouse oocytes (Dahl *et al*, 2016). We used the signals in ESCs as a reference so that ESCs had a scale factor of 1. We ranked the gene promoters by their H3K27ac signals. Then, for each stage, we chose the median signal of the top 3,000 promoters, dividing from the median signals of the top 3,000 promoters in ESCs to obtain the sale factor for that stage. The FPKM signals in each stage were multiplied by the scale factor to derive the scaled FPKM, which was used in all the downstream analyses.

For allelic analysis, we used SNPsplit (v0.5.0; Krueger & Andrews, 2016) to assign the mapped reads to maternal or paternal allele based on the SNPs information between B6D2F1and PWK/PhJ. The SNPs were downloaded from the Mouse Genomes Project (https://www.sanger.ac.uk/data/mouse‐genomes‐project/). The SNPs that were identical between C57BL/6J and DBA/2J while different from PWK/PhJ were used.

### Call acetylation domains

To call the broad acetylation domains in oocytes and zygotes, we split the genome into 1 kb bins. To call the narrow acetylation domains in 2 cell embryos, morulae, and ESCs, we split the genome into 500 bp bins. Then, the average adjusted FPKM of each bin was calculated using multiBigwigSummary in deeptools (v3.5.1). Bins with FPKM≥3 were used as acetylation domains. Domains within 2 × bin size from end to end were merged as final domains.

### RNA‐seq data analysis

For the raw sequencing reads of total RNA‐seq, the three leading nucleotides of read2 generated from TSO were removed. Then, the adaptors were removed with Trimmomatic (v0.39) if present. The cleaned reads were mapped to GRCm38 reference genome using STAR (v2.7.8a; Dobin *et al*, 2013). RSEM (v1.3.1; Li & Dewey, 2011) was employed to quantify the gene expression levels with the transcriptome alignments generated by STAR as input. For differential expression analysis, R package DESeq2 (v1.32.0; Love *et al*, 2014) was utilized with raw counts from RSEM as input. The significantly changed genes were identified with an adjusted *P*‐value cutoff of 0.05, fold change cutoff of 2, and mean FPKM cutoff of 1.

The repeat elements in the mouse genome were obtained from RepeatMasker (https://www.repeatmasker.org). For the repeat element expression analysis, we composed a pseudo‐genome with each type of repeats as a pseudo‐chromosome, by concatenating the sequences of all instances of this repeat type with 200 Ns as a spacer between any two sequences to compose the chromosome sequence. The corresponding gtf gene annotations were generated by treating the pseudo‐chromosome of each repeat type as a gene. Then, the RNA‐seq sequences were mapped to this pseudo‐genome by STAR and expression levels of repeats were quantified by RSEM.

### ATAC‐seq data analysis

For analyzing ATAC‐seq sequencing data, we used the ENCODE ATAC‐seq pipeline (v2.1.2, https://github.com/ENCODE‐DCC/atac‐seq‐pipeline). The optimal IDR replicated peaks were used for downstream analysis. ATAC‐seq peaks within 1 kb upstream or downstream of any known TSS were treated as promoter peaks, while ATAC‐seq peaks with a distance greater than 2.5 kb to TSS were treated as distal peaks.

### Motif enrichment

For TF motif enrichment analysis, HOMER (v4.11; Heinz *et al*, 2010) was employed with command findMotifsGenome.pl and default parameters.

### Hi‐C data analysis

The raw reads from Hi‐C were first trimmed with Trimmomatic (v0.39) to remove adaptors. Then, reads were mapped to GRCm38 reference genome by bwa mem (v0.7.17; Li & Durbin, 2010) with parameters ‐SP5M. The mapped reads were parsed by pairtools (v0.3.1, https://github.com/open2c/pairtools) to generate contact pairs. The contact pairs were filtered to remove duplicates, improper pairs, and pairs with a mapping quality less than 30. Contact matrixes were generated with cooler (v0.8.11; Abdennur & Mirny, 2020). For pile‐up analysis, we first enumerated all possible combinations between CBP/p300‐dependent ATAC‐seq peaks and ZGA genes promoters and stored them in bedpe format. Then, coolpup.py (v0.9.5; Flyamer *et al*, 2020) was used to calculate the pile‐up results with the bedpe file and the contact matrix at 10 kb resolution as input and parameters ‐‐features_format bedpe ‐‐nshifts 1 ‐‐seed 0.

### Defining zygote minor ZGA genes, early 2C minor ZGA genes, major ZGA genes, maternal decay genes, and PcG targets

Zygote minor ZGA genes were defined by comparing the transcriptome between DRB‐treated zygote (12 hpi) and control (DMSO‐treated) zygote, with fold change cutoff 2 and adjusted *P*‐value cutoff 0.05. Early 2‐cell minor ZGA genes were obtained by comparing the transcriptome between DRB‐treated early 2‐cell (20 hpi) and control (DMSO‐treated) early 2‐cell, with fold change cutoff 2 and adjusted *P*‐value cutoff 0.05. Major ZGA genes were defined as the up‐regulated genes between late 2‐cell and zygotes. Maternal decay genes were defined by combining the down‐regulated genes of zygote versus MII oocyte, early 2‐cell versus MII oocyte, late 2‐cell versus MII oocyte, and 4‐cell versus MII oocyte. For major ZGA genes and maternal decay genes, the fold change cutoff was 4 and the adjusted *P*‐value cutoff was 0.01. The PcG targets were defined as the genes with a promoter (± 2 kb of TSS) marked by H3K27me3 in inner cell mass and no expression (FPKM<0.1) in preimplantation embryos. All the gene lists were provided in Dataset EV1.

### Public data used in this study

The public datasets used in this study are summarized in Table EV1.

## Author contributions

**Meng Wang:** Conceptualization; data curation; software; formal analysis; validation; investigation; visualization; methodology; writing – original draft. **Zhiyuan Chen:** Conceptualization; data curation; software; formal analysis; validation; investigation; visualization; methodology; writing – review and editing. **Yi Zhang:** Conceptualization; resources; supervision; funding acquisition; investigation; project administration; writing – review and editing.

## Disclosure and competing interests statement

The authors declare that they have no conflict of interest.

## Supporting information



Expanded View Figures PDF

Table EV1

Dataset EV1

Dataset EV2

Dataset EV3

Dataset EV4

Dataset EV5

PDF+

## Data Availability

The datasets (and computer code) produced in this study are available in the following databases: CUT&RUN data, RNA‐Seq data, and ATAC‐seq data: Gene Expression Omnibus GSE207222 (https://www.ncbi.nlm.nih.gov/geo/query/acc.cgi?acc=GSE207222).
