# Supplementary material for: CBP/p300 and HDAC activities regulate H3K27 acetylation dynamics and zygotic genome activation in mouse preimplantation embryos
Source: EMBO J. 2022 Oct 10;41(22):e112012. doi: 10.15252/embj.2022112012 (PMC9670200; doi:10.15252/embj.2022112012)
Supplement: Supplementary file 1 — Expanded View Figures PDF [file EMBJ-41-e112012-s003.pdf]

Expanded View Figures

Figure EV1. Dynamics of H3K27ac in mouse oocytes and early embryos.

A Spearman correlation of H3K27ac CUT&RUN replicates and correlation with available public data. The public H3K27ac data were generated using the μChIP–seq method (Dahl *et al*, 2016). GV—germinal vesicle oocyte; FGO—fully grown oocyte; 2C—2-cell embryo.
B Scale factors for H3K27ac FPKM at different stages. The scale factors at top 3,000 promoters were used.
C H3K27ac domain bases distribution at promoter, exon, intron, and intergenic regions for each stage.
D Dynamic changes of H3K27ac from early 2-cell to morula stage and ESC. C: domain center.
E Correlations between gene density and H3K27ac signals for each stage.
F H3K27ac signal enrichment around TSS of genes with CGI or non-CGI promoters. CGI: CpG island.
G Enrichment of H3K27ac at ERVL retrotransposons at different stages.
H Genome browser view showing different dynamics of H3K27ac and H3K9ac from zygotes to late 2-cell embryos.

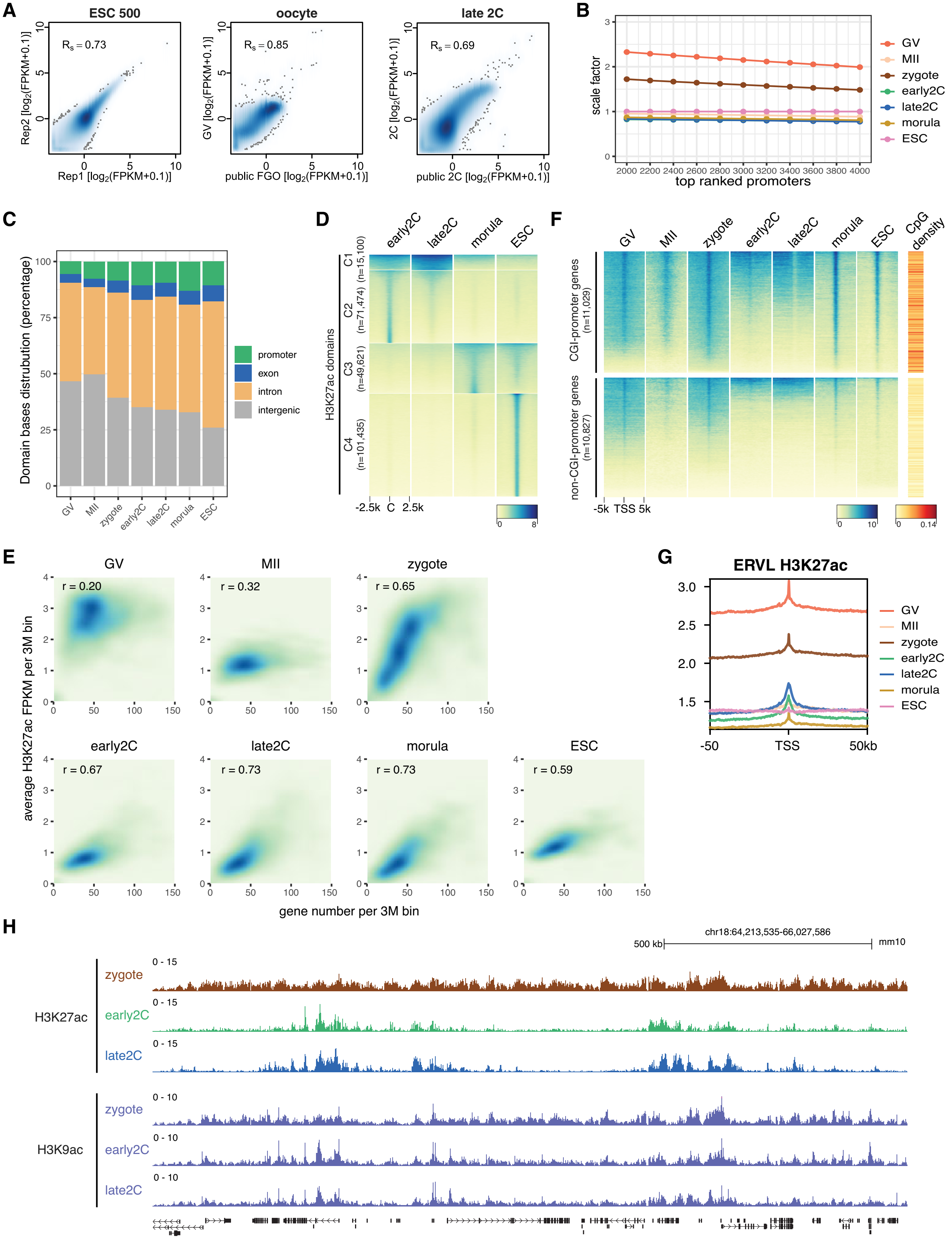

Figure EV1.

 © 2022 The Authors

Figure EV2. The allele-specific level of H3K27ac.

A Immunostaining images of fertilized 1-cell embryos stained with anti-H3K27me3 and anti-H3K27ac at indicated time points post fertilization. M—maternal DNA; P—paternal DNA; Pb—polar body. Scale bar: 20 μm.

B Allele-specific enrichment of H3K27ac signals at ERVL retrotransposons at different stages. M—maternal allele; P—paternal allele.

C Comparison of H3K27ac signals at paternal imprinting control regions (ICR, $n = 4$) between the maternal allele and paternal allele at different stages. The *P*-values were derived from one-sided *t*-test. The list of paternal ICRs were retrieved from (Xie *et al*, 2012).

D Comparison of H3K27ac signals at major ZGA genes promoters ($n = 2,773$, $\pm$ 2 kb of TSS) between the maternal allele and paternal allele at different stages. The *P*-values were derived from two-sided *t*-test.

Data information: For boxplots in (C) and (D), the central band represents the median. The lower and upper edges of the box represent the first and third quartiles, respectively. The whiskers of the boxplot extend to 1.5 times interquartile range (IQR).

© 2022 The Authors

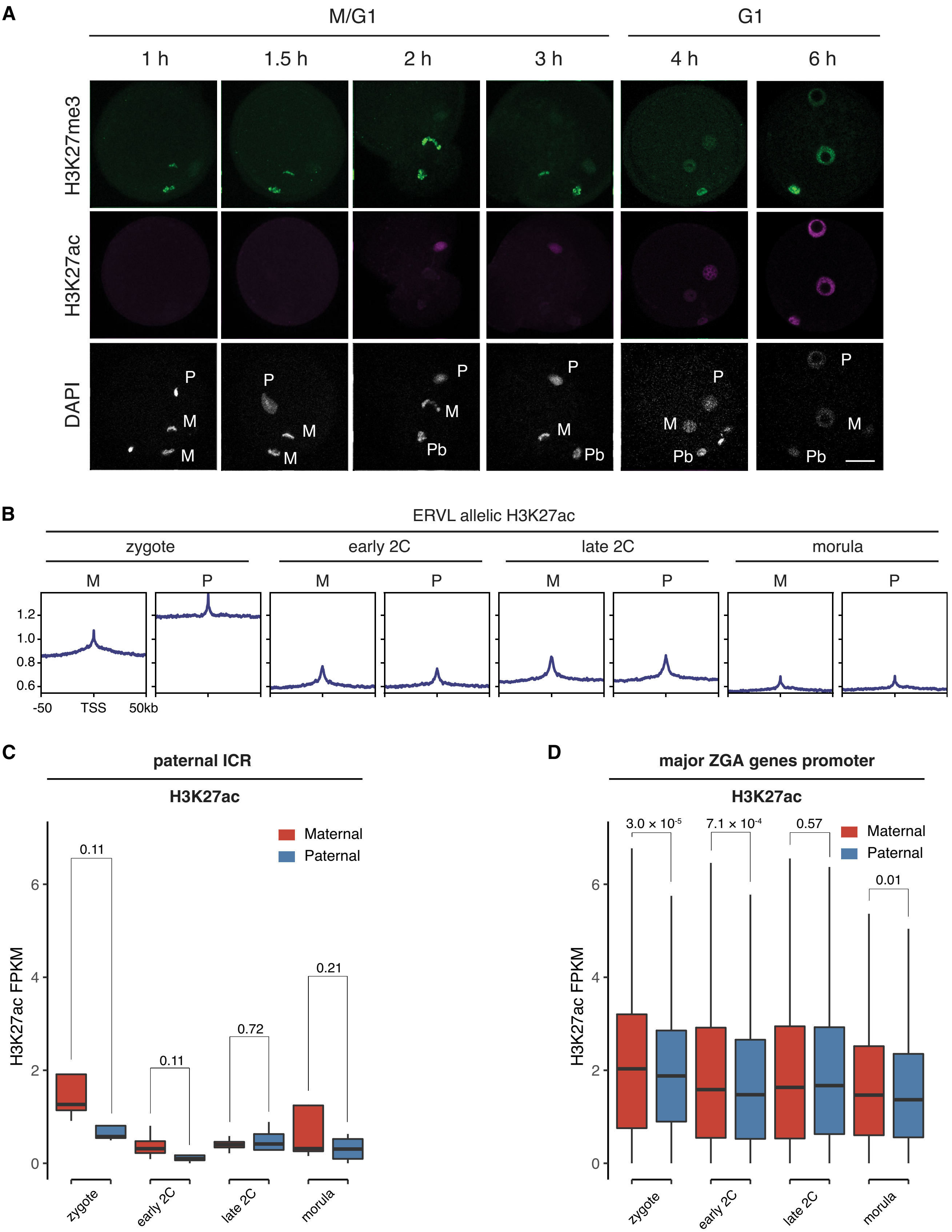

Figure EV2.

 © 2022 The Authors

Figure EV3. Transcriptome comparison of CBP/p300 inhibition versus control.

A Expression level dynamics of *Ep300* (p300) and *Crebbp* (CBP) in MII oocyte and preimplantation embryos.
B Determination of optimal A-485 concentration to inhibit CBP/p300 by H3K27ac immunostaining. The embryos (3 biological replicates in each condition) were treated with A-485 starting at 4 hpi, and images were taken at 7 hpi. Scale bar: 20 μm.
C Immunostaining quantification of H3K27ac relative intensities in Fig 4B (6–12 biological replicates in each condition).
D Pearson correlation of RNA-seq replicates at zygote, early 2-cell, and late 2-cell stage for control (DMSO-treated) and CBP/p300 inhibition (A-485-treated).
E Gene Ontology (GO) enrichment for the down-regulated genes at early 2-cell stage after A-485 treatment.
F Scatter plot showing the expression level changes of repeat elements after CBP/p300 inhibition.

Data information: For boxplots in (B) and (C), the central band represents the median. The lower and upper edges of the box represent the first and third quartiles, respectively. The whiskers of the boxplot extend to 1.5 times interquartile range (IQR).

© 2022 The Authors

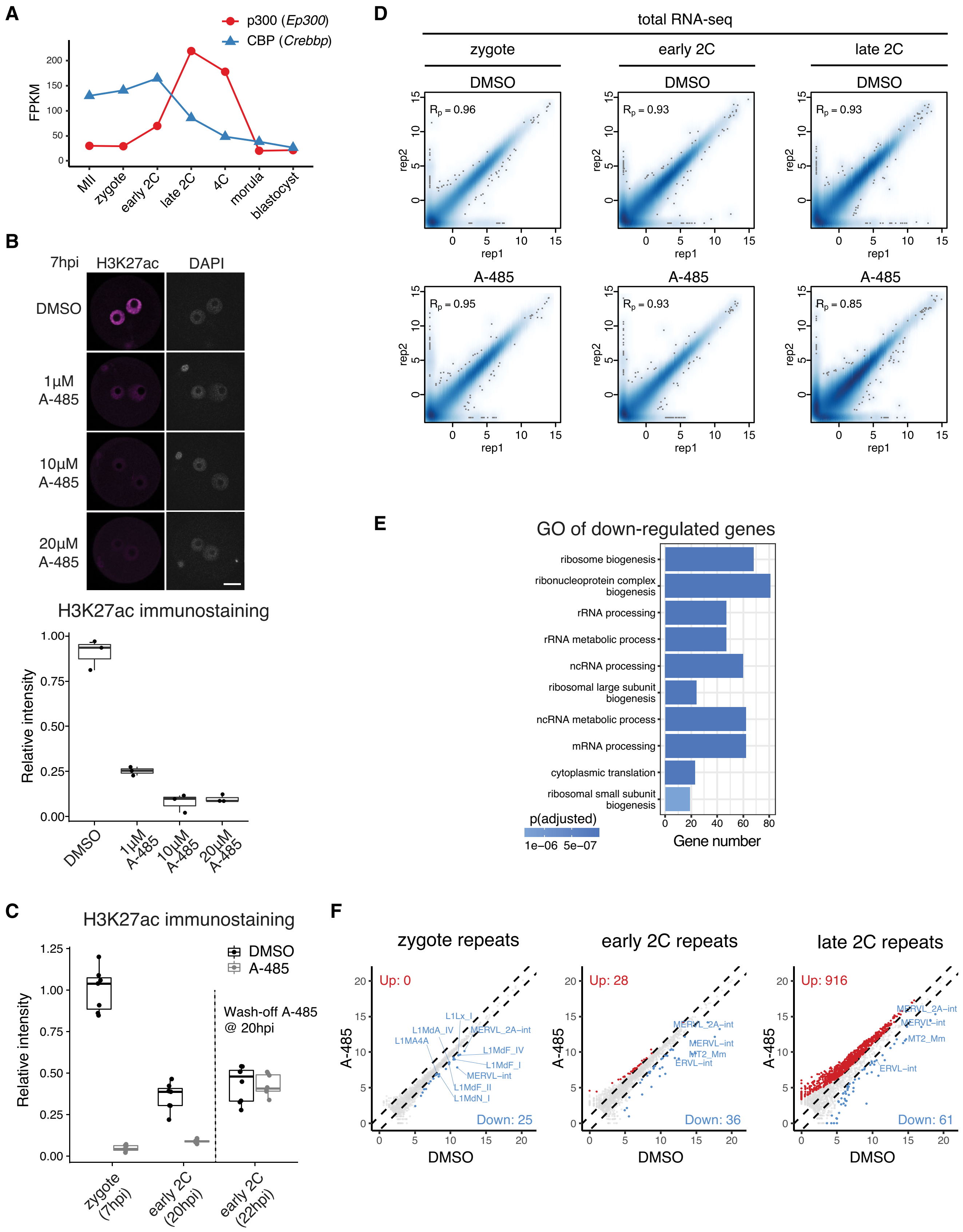

Figure EV3.

 © 2022 The Authors

Figure EV4. Chromatin accessibility comparison of CBP/p300 inhibition versus control.

A Pearson correlation of ATAC-seq replicates for DMSO-treated and A-485-treated late 1-cell and early 2-cell.
B ATAC-seq signal comparison for control and A-485-treated late 1-cell. C—peak center.
C ATAC-seq signal comparison for control and minor ZGA inhibition (treated with α-Amanitin) at early 2-cell stage. The ATAC-seq peaks were the same as in Fig 5A. The ATAC-seq data of control early 2-cell and α-Amanitin-treated early 2-cell were from GEO with accession GSM1933921, GSM1933922, GSM1933923, and GSM2108702 (Wu *et al*, 2016).
D Gene Ontology (GO) enrichment for the nearest genes of the ATAC-seq peaks that were lost under A-485 treatment in early 2-cell, using GREAT analysis (McLean *et al*, 2010).
E Distance to TSS for the ATAC-seq peaks that were lost after A-485 treatment in early 2-cell embryos.
F Pile-up of Hi-C contacts at 2-cell stage between CBP/p300-dependent distal ATAC-seq peaks (x-axis) and randomly selected non-ZGA genes TSS regions (n = 2,773, matched chromosome distribution with major ZGA genes).

© 2022 The Authors

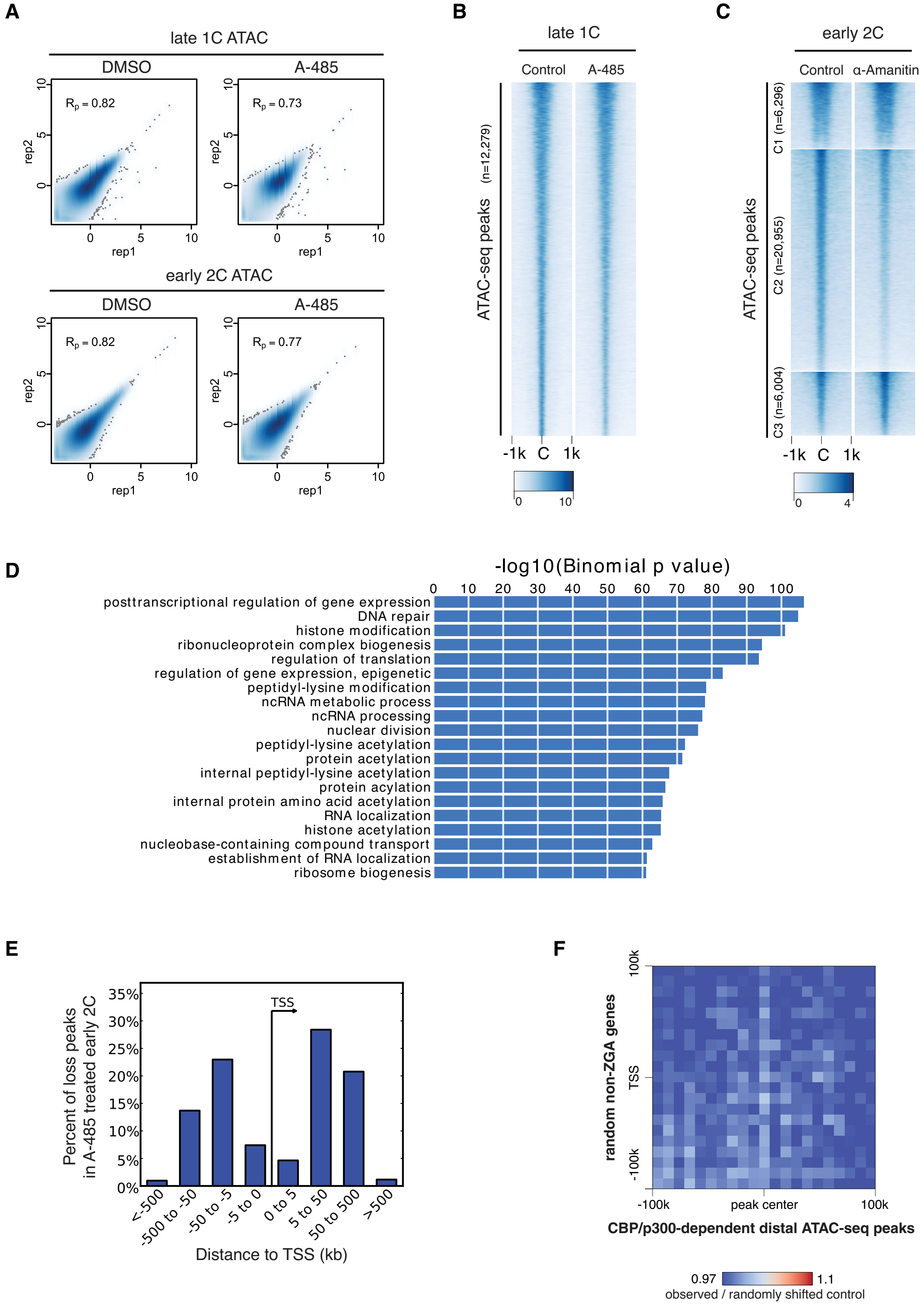

Figure EV4.

 © 2022 The Authors

Figure EV5. Transcriptome comparison of HDAC inhibition versus control.

A Expression dynamics of HDAC genes in mouse MII oocyte and preimplantation embryos.
B Immunostaining quantification of H3K27ac relative intensities in Fig 6B (4–16 biological replicates in each condition).
C Pearson correlation of RNA-seq replicates at late 2-cell stage for control (DMSO-treated) and HDAC inhibition (TSA-treated).
D Heatmap showing the down-regulated genes after TSA treatment in late 2-cell embryos and the corresponding H3K27ac dynamics at the promoters of these down-regulated genes.
E Gene Ontology (GO) enrichment analysis of the down-regulated genes after HDAC inhibition.
F Scatter plot showing the impact of HDAC inhibition on expression levels of major ZGA genes.
G Scatter plot showing the impact of HDAC inhibition on expression levels of maternal decay genes.

Data information: For boxplots in (B), the central band represents the median. The lower and upper edges of the box represent the first and third quartiles, respectively. The whiskers of the boxplot extend to 1.5 times interquartile range (IQR).

© 2022 The Authors

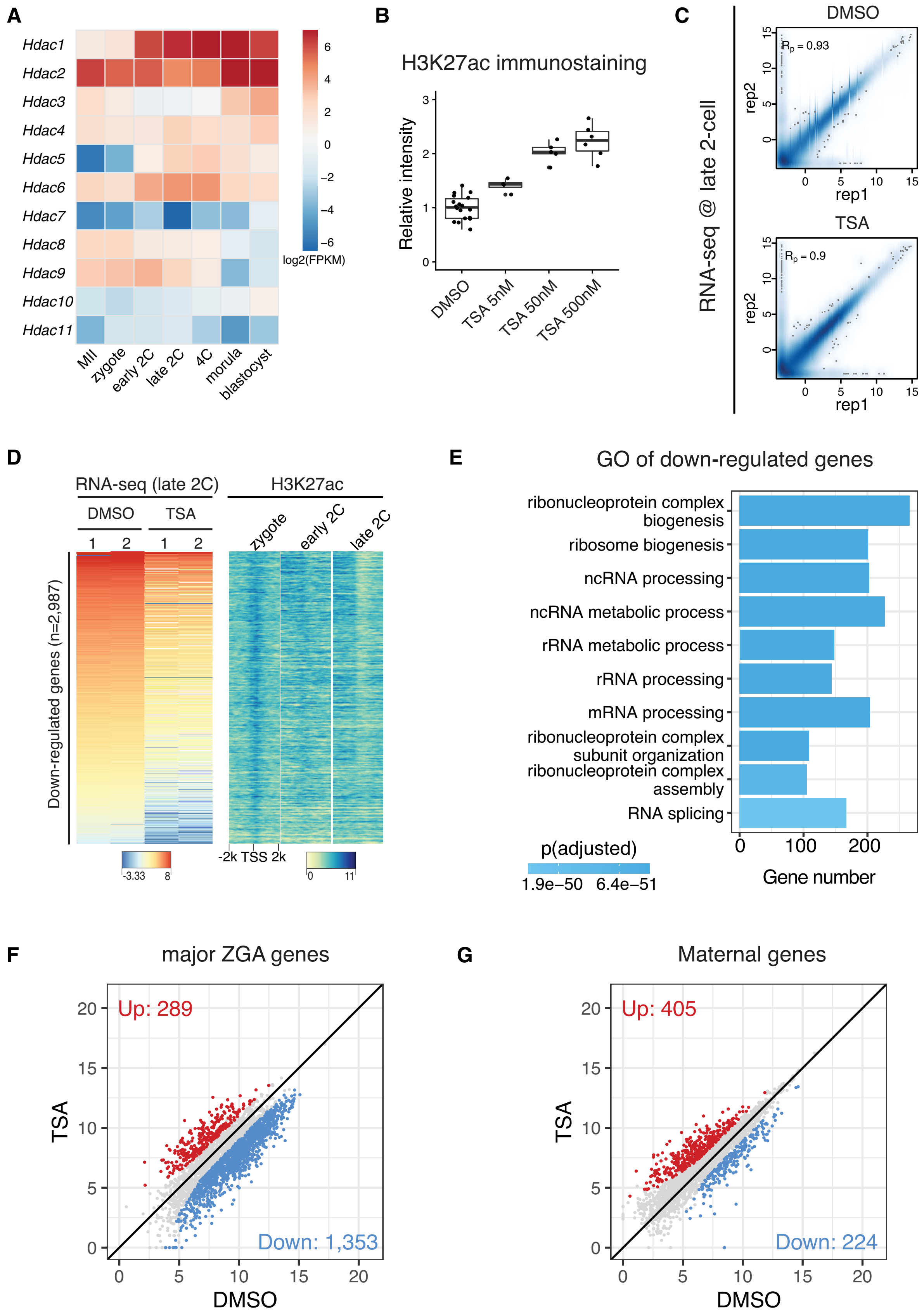

Figure EV5.

© 2022 The Authors
