# Supplementary material for: CBP/p300 and HDAC activities regulate H3K27 acetylation dynamics and zygotic genome activation in mouse preimplantation embryos
Source: EMBO J. 2022 Oct 10;41(22):e112012. doi: 10.15252/embj.2022112012 (PMC9670200; doi:10.15252/embj.2022112012)
Supplement: Supplementary file 9 [file EMBJ-41-e112012-s009.pdf]

THE EMBO JOURNAL

CBP/p300 and HDAC activities regulate H3K27 acetylation dynamics and zygotic genome activation in mouse preimplantation embryos

Meng Wang, Zhiyuan Chen, and Yi Zhang
DOI: 10.15252/embj.2022112012

Corresponding author(s): Yi Zhang (yzhang@genetics.med.harvard.edu)

Review Timeline:

Submission Date:	29th Jun 22
Editorial Decision:	3rd Aug 22
Revision Received:	30th Aug 22
Editorial Decision:	20th Sep 22
Revision Received:	20th Sep 22
Accepted:	23rd Sep 22

Editor: Stefanie Boehm

Transaction Report:

(Note: With the exception of the correction of typographical or spelling errors that could be a source of ambiguity, letters and reports are not edited. Depending on transfer agreements, referee reports obtained elsewhere may or may not be included in this compilation. Referee reports are anonymous unless the Referee chooses to sign their reports.)

1st Editorial Decision 3rd Aug 2022

Thank you again for submitting your manuscript (EMBOJ-2022-112012) to The EMBO Journal. As mentioned, one referee report was delayed, but we have now received the three reports below on your study. We discussed the comments amongst the editorial team and have decided to invite you to prepare and submit a revised manuscript.

As you will see, the reviewers are overall positive and appreciate the findings and their interest to the field. However, they do raise several points, which will have to be addressed in the revised version of the manuscript. In particular, please clarify the timing of A-485 treatment and contribution of minor ZGA (ref #2- point 4; ref #3- point 1, 2), as well as the ZGA gene set definition (ref #2- point 2; ref #3- point 7). In addition, both referee #1 (point 5) and referee #3 (additional comment 1) are concerned about the H3K27ac IF staining at the polar body, please respond to these concerns and provide additional data to exclude issues with the experimental approach if needed. Referee #3 also raises the concern that the cell cycle arrest induced by TSA treatment may have an additional effect (point 3). Please consider this point carefully and provide experimental data to differentiate effects and/or revise the manuscript to discuss this. In addition, please also carefully consider all other referee comments and revise the manuscript and figures as needed. When submitting the revised manuscript please remember to include a detailed response to each comment and that the revised manuscript should then fulfill all EMBO Journal formatting requirements (please see below and: https://www.embopress.org/page/journal/14602075/authorguide#submissionofrevisions)

Referee #1:

In this manuscript, Meng et al. profiles H3K27ac dynamics during preimplantation development and provide evidence suggesting that both CBP/p300 and HDACs are essential for ZGA and preimplantation development. The study is informative, well-constructed, and offers conceptual advances in epigenetic regulation on embryonic development. On the other hand, several statements in the manuscript lack experimental evidence and merit further analysis.

1, CBP/p300 affects distal open chromatin regions according to Fig. 5C. The authors suggest that these regions may serve as enhancers of ZGA genes based on Hi-C analysis. To further verify the hypothesis, it is crucial to examine whether the genes related to these "enhancers" are transcriptionally altered upon CBP/p300 inhibition.

2, H3K9ac dynamics in preimplantation development have been profiled. It would be interesting to compare the deposition and function of H3K9ac and H3K27ac during the preimplantation development.

3, CBP/p300 and HDACs affect multiple histone acetylation. As the H3K27ac and H3K9ac profiling have been performed, I recommend evaluating how H3k27ac and H3k9ac contribute to the effect of CBP/p300 and HDACs in the preimplantation development and ZGA.

4, The HDAC family contains multiple proteins. Could the author detect the expression of these HDACs in preimplantation embryos and speculate which HDAC plays a significant role in ZGA and preimplantation development?

5, In Fig. 1A, although the MII oocytes had very low H3K27ac level, I noticed clear H3K27ac signal in the polar body. Is that suggest quick H3K27ac deposition after fertilization but before PB2 emission? I hope the authors have an explanation to this result.

6, Authors need to follow the guidelines of gene symbols, protein symbols, RNA symbols: gene and RNA symbols should be italicized (also in the figures), and protein abbreviations should be in capital letters.

7, CBP/P300 inhibitor A-485 and HDAC inhibitor TSA lead to downregulation and upregulation of H3K27ac in early embryos, respectively. But it will be interesting to know if H3K27me4 levels are also changed in opposite trends after these drug treatments.

Referee #2:

EMBOJ-2022-112012 review

Wang et al. describe the dynamics of H3K27ac in oocytes and mouse preimplantation embryos during the maternal-to-zygotic transition (MZT). They identify three waves of H2K37ac reprogramming, with large, broad non-canonical H3K27ac domains present in hyperacetylated GV oocytes, loss of most H3K27 acetylation in hypoacetylated MII oocytes, and regain of broad H3K27ac in again hyperacetylated zygotes. After zygotic cleavage, in early and late 2C embryos, canonical patterns and H3K27ac peaks are established for the first time. However, before that, in the zygote, the authors noted that broad H3K27ac domains correlate with open chromatin, and may prime genomic regions for zygotic genome activation (ZGA) and upregulation of ZGA associated retrotransposons. The authors also suggest a functional role for CBP/p300 in establishment of these regions and opening up chromatin, and propose HDACs mediate the transition to more canonical H3K27ac patterns during ZGA and prevent premature developmental gene expression. The results in this manuscript are of interest to the fields of genome activation and epigenetic remodelling during preimplantation development. The claims are mostly supported by functional and mechanistic data. Especially, the finding that potential ZGA gene enhancers might already be primed in the zygote is interesting, but might need a bit more analyses or data for its support. A few other points still need to be addressed.

Can paternal ICRs be analysed as well for their H3K27ac levels (despite the too low numbers)? As H3K27ac levels are higher on the paternal allele in general, it is probably not too surprising that maternal ICRs are higher H3K27 acetylated on the paternal allele as well (Figure 3E and F). Similarly, as H3K27me3 is higher on the maternal allele in zygotes, it does not seem too surprising that H3K27me3 is higher in 'H3K27me3 dependent imprinting gene promoters' on the maternal allele either. Can the authors analyse the levels at other regions as controls (not ICRs) as well, or discuss the results more with respect to the later stages, where the global differences seem lost but the differences at the imprinted regions maybe remain?

As the authors previously stated that major ZGA occurs at the late 2C (not early 2C) and they include both gene lists "early 2C ZGA genes" and "major ZGA genes" in their methods, they need to be more specific in the text and figure legends with the use of "ZGA genes" - which lists they exactly use for their analyses. For example, page 10, main text: "Comparative whole transcriptome analysis indicated that most of the early 2C? ZGA genes were down-regulated after CBP/p300 inhibition." And page 11 - middle paragraph, last sentence. "CBP/p300-dependent distal open chromatin regions at early 2-cell stage may function as enhancers of ZGA genes". Are these "major ZGA genes" or "early 2C ZGA genes"? - please specify. Also, legend Figure 5E: "ZGA gene TSS" - please specify.

Page 10: The results of Figure EV4E and EV4F are not included in the text. It would be good to at least mention the GO term enrichment analysis results of downregulated genes in early 2C embryos after A-485 treatment - Figure EV4F (ribosome biogenesis etc).

When were the embryos harvested after the A-485 treatment - at 20h or 22h after the washout? Can the authors please write in text or indicate in scheme in Figure 4A (similar for TSA treatment - when were the embryos harvested, Figure 6A)? Can the authors please provide a brief rational as to why they choose to analyse early 2C embryos and not late 2C embryos for their inhibitor experiments? Would they expect major ZGA in late 2C embryos to be similarly disturbed upon transient A-485 treatment, or would they expect early 2C ZGA genes to be upregulated after wash-out of the inhibitor at later stages, for example in late 2C embryos?

Minor Comments:
• Please provide row numbers in your manuscript upon resubmission.
• Typo abstract: "Specifically, CBP/p300 deposits broad H3K27ac domains in zygotes and opens condensed chromatin at putative enhancers"
• Figure EV1: Please provide info on the abbreviation 'FGO', and specify if the 'public FGO' data that was used by Dahl et al., 2016 is biologically different to the 'GV oocyte' data that as used in this study. Are these the same stages, I assume so?
• Typo Page 4: Depletion of HDAC1/2 results in early embryonic lethality. [...] how their activities contributione to ZGA remain largely unknown.
• Typo Page 10: ATAC-seq peaks that were lost after BFP/p300 inhibition (2x on page)
• Typo Figure EV1C: Domain bases distribution
• Methods: Please provide at least a few details about the immunostaining in the methods section (permeabilization and fixation buffer, length etc) instead of just giving a reference.

Referee #3:

Histone H3K27ac is associated with the establishment of enhancers/promoters to activate transcription. Wang & Chen et al.

used CUT&RUN to reveal the allelic H3K27ac landscape during the maternal-to-zygotic transition. The results suggest that p300/CBP-mediated H3K27ac is required for ZGA and early embryonic development. HDAC activity prevents premature expression of developmental genes. In general, this is a strong manuscript with well designed experiments. However, some important claims are not fully supported by the data and would be strengthened by additional data and analysis as detailed below. Another weakness is that the results rely entirely on chemical inhibition and have not been corroborated by other perturbations.

Major comments:

1. Transient inhibition of CBP/p300 by A-485 from 4-20 hpf caused a 2-cell arrest and downregulation of early 2C ZGA genes (Fig. 4). Since minor ZGA is essential for subsequent major ZGA and development beyond 2-cell stage (Abe et al., 2018, PNAS), an alternative interpretation of this result is that H3K27ac regulates expression of minor ZGA genes and the failure to activate these affects 2C ZGA genes. To distinguish between these possibilities, RNA-seq of zygotes treated with A-485 needs to be performed.

2. Abe et al., 2018 also showed DRB treatment from minor ZGA showed loss of H3K4me3 in 2-cell embryos. It is thus necessary to test whether the canonical H3K27ac pattern in 2-cell embryos is dependent on minor ZGA.

3. The interpretation of the HDAC inhibitor experiments is complicated by TSA inducing cell cycle arrest via p21 (Hrgovic et al., 2016). The early treatment (0-20 h) might not have any effect because checkpoints are less active in zygotes. The 8-28 h treatment will overlap with G1 phase of 2C embryos. It is therefore important to delineate the timing of arrest, i.e. does it occur in G1 or G2 phase? If arrest occurs in G1 phase and major ZGA typically occurs during and after S phase, then how can the authors disentangle a direct from an indirect effect on ZGA transcription?

4. The observation that H3K27 is transiently hypoacetylated in MII oocytes both by by immunofluorescent staining and to some extent CUT&RUN is interesting. To exclude that the H3K27ac domain decrease (Fig. 1C) is not due to chromatin inaccessibility for Tn5 in condensed chromosomes, CUT&RUN should be performed with another histone modification such as H3K9ac or H3S10ph to demonstrate that histone PTMs are readily detectable at this stage using this method.

5. There appears to be hardly any detectable H3K27ac by immunostaining of MII oocytes (Fig. 1A) yet CUT&RUN profiling of the same stage oocytes shows a clear enrichment of H327ac (Fig. 1B). The authors should clarify this apparent discrepancy.

6. It has recently been reported that H3K27ac is not essential for gene derepression and de novo activation of genes during some cell fate decisions (Sankar et al., Nature 2022) and that PTMs other than H2K27ac are involved in mediating CBP/p300 function. The authors should discuss their results in light of this key finding.

7. How were ZGA genes defined and what percent of these are significantly downregulated upon CBP/p300 inhibition?

8. The interpretation of all experiments using A-485 critically depends on the specifity of the inhibitor. What is the evidence that A-485 is specific at the concentration used to treat embryos?

9. Is there any correlation between the 416 downregulated ZGA genes inn A-485 treated embryos (Fig. 4E) and those that have enriched contacts with CBP/p300-dependent open chromatin (Fig. 5E)?

10. A pile-up Hi-C analysis of CBP/p300-dependent distal open chromatin regions and shuffled sets of non-ZGA genes should be performed as a control to determine whether the higher contacts are specific to ZGA genes.

11. If H3K27ac is priming major ZGA genes, then one would expect that H3K27ac is also enriched at TSS of 2C embryos, yet the signal appears to be lower (Fig. 1F). How can this be explained?

Additional comments:
1. The H3K27ac signal is clearly stronger in the nuclei of zygotes compared to 2C embryos (Fig 1A). However, it is puzzling to see that a similar decrease is visible for the polar body, raising a possible concern that the immunofluorescent detection was overall less effective in 2C embryos compared to zygotes.

2. Previous work showing that p300 perturbation affects H3K27me3 more at enhancers than promoters (Martire et al., 2020) be referenced.

We thank the reviewers for their careful and constructive comments. We provide point-by-point responses below.

Referee #1:

In this manuscript, Meng et al. profiles H3K27ac dynamics during preimplantation development and provide evidence suggesting that both CBP/p300 and HDACs are essential for ZGA and preimplantation development. The study is informative, well-constructed, and offers conceptual advances in epigenetic regulation on embryonic development. On the other hand, several statements in the manuscript lack experimental evidence and merit further analysis.

We thank the reviewer for the overall positive comments on this study.

1. CBP/p300 affects distal open chromatin regions according to Fig. 5C. The authors suggest that these regions may serve as enhancers of ZGA genes based on Hi-C analysis. To further verify the hypothesis, it is crucial to examine whether the genes related to these "enhancers" are transcriptionally altered upon CBP/p300 inhibition.

We thank the reviewer for the suggestion. Based on the Hi-C data, we showed these putative enhancers have higher than expected contacts with major ZGA genes. Previously we showed that the early 2C minor ZGA genes are down-regulated upon CBP/p300 inhibition (Fig. 4). Thus, we believe major ZGA genes would also be down-regulated. To directly show this and follow the reviewer’s suggestion, we performed RNA-seq of late 2-cell after CBP/p300 inhibition and examined how major ZGA genes would change. We found that most major ZGA genes are down-regulated after CBP/p300 inhibition (Fig. 4F). We also showed that the CBP/p300 activity is critical for minor ZGA, and for opening the distal chromatin regions at early 2-cell (Fig. 4 and 5), both of which are important for major ZGA. The minor ZGA is known to regulate major ZGA (PMID: 29967139). Collectively, our data support the notion that the CBP/p300-dependent distal opening elements may function as enhancers to regulate the major ZGA genes.

2. H3K9ac dynamics in preimplantation development have been profiled. It would be interesting to compare the deposition and function of H3K9ac and H3K27ac during the preimplantation development.

We reanalyzed the public H3K9ac profiling data in mouse zygotes, early 2-cell and late 2-cell embryos. In zygotes, both H3K27ac and H3K9ac showed broad distribution (Fig. EV2). Further examination suggested that H3K27ac and H3K9ac domains are colocalized in zygotes (Fig. 2D), although they are deposited by different enzymes, indicating a hyperacetylation state of multiple histones sites in zygotes. However, the reprogramming dynamics of H3K27ac and H3K9ac are totally different. While most H3K27ac domains were deacetylated from zygotes to early 2-cell embryos, H3K9ac remained largely unchanged from zygote to early 2-cell and late 2-cell stages (Fig. EV2). We describe this result from line 203 to line 210.

3. CBP/p300 and HDACs affect multiple histone acetylation. As the H3K27ac and H3K9ac profiling have been performed, I recommend evaluating how H3k27ac and H3k9ac contribute to the effect of CBP/p300 and HDACs in the preimplantation development and ZGA.

We showed HDAC activities are critical for proper ZGA and normal development (Fig. 6), which coincide with the deacetylation of H3K27ac from zygote to early 2-cell. In contrast, the H3K9ac remained largely unchanged from zygote to early 2-cell (Fig. EV2), suggesting they may have different functions in regulating ZGA and preimplantation development. Since these two modifications are deposited by different enzymes, and this manuscript is mainly focused on H3K27ac and related enzymes, we decided not to exam the relationship between H3K9ac and CBP/p300. But we agree with the reviewer that it might worth to investigate the cooperative and different functions of different histone acetylation modifications like H3K27ac, H3K9ac, H4K16ac etc. during mouse preimplantation development in future studies.

4. The HDAC family contains multiple proteins. Could the author detect the expression of these HDACs in preimplantation embryos and speculate which HDAC plays a significant role in ZGA and preimplantation development?

We thank the reviewer for the suggestion. We checked the expression level of the HDAC genes (Fig. EV6A), which showed that HDAC1 and HDAC2 are highly expressed in early 2-cell embryos, which coincide with the extensive deacetylation at early 2-cell stage. This indicates that HDAC1/2 may be the key factors in the process. Consistently, we noticed a recent paper reporting HDAC1/2 are required to safeguard ZGA in mice (PMID: 35575026). We cited this paper and added discussion in line 433 to line 437.

5. In Fig. 1A, although the MII oocytes had very low H3K27ac level, I noticed clear H3K27ac signal in the polar body. Is that suggest quick H3K27ac deposition after fertilization but before PB2 emission? I hope the authors have an explanation to this result.

To resolve when the zygote genome acquires H3K27ac after fertilization, we performed time-course immunostaining of H3K27ac (Fig. EV3A), which showed that the paternal allele *de novo* established the H3K27ac as early as 2 hours post *in vitro* fertilization (hpi), while the maternal allele began to obtain H3K27ac from 4 hpi. The PB2 also acquires H3K27ac from 4 hpi (after its exclusion), in parallel with the maternal allele, suggesting the cytoplasm in polar body also contains CBP/p300 enzymatic activity.

6. Authors need to follow the guidelines of gene symbols, protein symbols, RNA symbols: gene and RNA symbols should be italicized (also in the figures), and protein abbreviations should be in capital letters.

We thank the reviewer for the suggestion. We have changed the symbols accordingly.

7. CBP/P300 inhibitor A-485 and HDAC inhibitor TSA lead to downregulation and upregulation of H3K27ac in early embryos, respectively. But it will be interesting to know if H3K27me3 levels are also changed in opposite trends after these drug treatments.

Following the reviewer's suggestion, we performed immunostaining of H3K27me3 after A-485 / TSA treatment (Fig. R1). Depletion or increase H3K27ac by inhibitor treatments did not alter

H3K27me3 levels toward the opposite direction. Interestingly, inhibition of CBP/p300 caused slight decrease of H3K27me3 signals. It would be interesting to study how H3K27ac and H3K27me3 are co-regulated in early embryos in future studies.

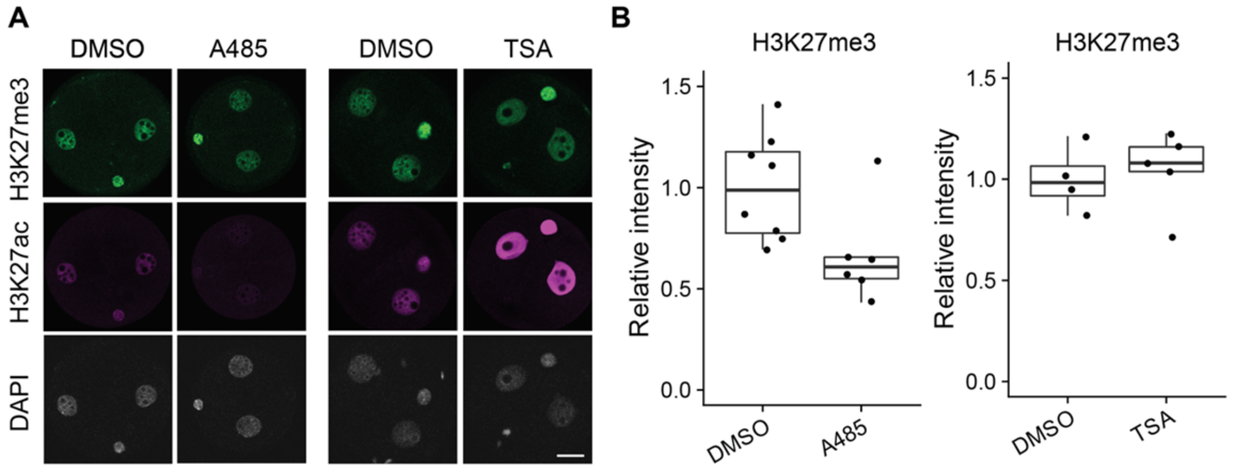

Figure R1. A: Immunostaining of H3K27me3 and H3K27ac in 2-cell embryos treated with DMSO, A485 (10 μM, 4-22 hpi), or TSA (50nM, 0-20hpi). Scale bar: 20 μm. **B**: Immunostaining quantification of H3K27me3 relative intensities in panel A.

Referee #2:

Wang et al. describe the dynamics of H3K27ac in oocytes and mouse preimplantation embryos during the maternal-to-zygotic transition (MZT). They identify three waves of H2K37ac reprogramming, with large, broad non-canonical H3K27ac domains present in hyperacetylated GV oocytes, loss of most H3K27 acetylation in hypoacetylated MII oocytes, and regain of broad H3K27ac in again hyperacetylated zygotes. After zygotic cleavage, in early and late 2C embryos, canonical patterns and H3K27ac peaks are established for the first time. However, before that, in the zygote, the authors noted that broad H3K27ac domains correlate with open chromatin, and may prime genomic regions for zygotic genome activation (ZGA) and upregulation of ZGA associated retrotransposons. The authors also suggest a functional role for CBP/p300 in

establishment of these regions and opening up chromatin, and propose HDACs mediate the transition to more canonical H3K27ac patterns during ZGA and prevent premature developmental gene expression. The results in this manuscript are of interest to the fields of genome activation and epigenetic remodeling during preimplantation development. The claims are mostly supported by functional and mechanistic data. Especially, the finding that potential ZGA gene enhancers might already be primed in the zygote is interesting, but might need a bit more analyses or data for its support. A few other points still need to be addressed.

We thank the reviewer for the nice summary.

1. Can paternal ICRs be analyzed as well for their H3K27ac levels (despite the too low numbers)? As H3K27ac levels are higher on the paternal allele in general, it is probably not too surprising that maternal ICRs are higher H3K27 acetylated on the paternal allele as well (Figure 3E and F). Similarly, as H3K27me3 is higher on the maternal allele in zygotes, it does not seem too surprising that H3K27me3 is higher in 'H3K27me3 dependent imprinting gene promoters' on the maternal allele either. Can the authors analyze the levels at other regions as controls (not ICRs) as well, or discuss the results more with respect to the later stages, where the global differences seem lost but the differences at the imprinted regions maybe remain?

Following this reviewer's suggestion, we analyzed the allele-specific H3K27ac levels for paternal ICRs (Fig. EV3C). We found that the maternal allele shows higher levels of H3K27ac than the paternal allele, despite paternal allele has higher H3K27ac levels globally. The p-values are not significant probably due to the small number of paternal ICRs (n = 4). As controls, we further analyzed the allele-specific H3K27ac levels for major ZGA genes promoters (Fig. EV3D). For major ZGA genes promoters, the two alleles have comparable levels of H3K27ac at late 2-cell stage as well as similar H3K27ac levels at other stages. We described these results in line 252 to 258.

2. As the authors previously stated that major ZGA occurs at the late 2C (not early 2C) and they include both gene lists "early 2C ZGA genes" and "major ZGA genes" in their methods, they

need to be more specific in the text and figure legends with the use of "ZGA genes" - which lists they exactly use for their analyses. For example, page 10, main text: "Comparative whole transcriptome analysis indicated that most of the early 2C? ZGA genes were down-regulated after CBP/p300 inhibition." And page 11 - middle paragraph, last sentence. "CBP/p300-dependent distal open chromatin regions at early 2-cell stage may function as enhancers of ZGA genes". Are these "major ZGA genes" or "early 2C ZGA genes"? - please specify. Also, legend Figure 5E: "ZGA gene TSS" - please specify.

We thank the reviewer for the suggestion. There are two waves of ZGA in mouse embryos – minor ZGA occurring at late 1-cell to early 2-cell stage and major ZGA at late 2-cell stage. To dissect the fine scale impact of CBP/p300 inhibition, we further separated minor ZGA genes into zygote minor ZGA genes (n = 30, Dataset EV1) and early 2-cell minor ZGA genes (n = 558, Dataset EV1). We clarified zygote minor ZGA genes, early 2C minor ZGA genes and major ZGA genes in the text and figure legends.

3. Page 10: The results of Figure EV4E and EV4F are not included in the text. It would be good to at least mention the GO term enrichment analysis results of downregulated genes in early 2C embryos after A-485 treatment - Figure EV4F (ribosome biogenesis etc).

We thank the reviewer for the suggestion. We have now described these panels in the revised manuscript.

4. When were the embryos harvested after the A-485 treatment - at 20h or 22h after the washout? Can the authors please write in text or indicate in scheme in Figure 4A (similar for TSA treatment - when were the embryos harvested, Figure 6A)? Can the authors please provide a brief rational as to why they choose to analyze early 2C embryos and not late 2C embryos for their inhibitor experiments? Would they expect major ZGA in late 2C embryos to be similarly disturbed upon transient A-485 treatment, or would they expect early 2C ZGA genes to be upregulated after wash-out of the inhibitor at later stages, for example in late 2C embryos?

We thank the reviewer for the suggestion. We clarified the time point of embryo collection in Fig. 4A and Fig. 6A. Since we want to investigate the earlier and direct effect of inhibitor treatment, we choose early 2-cell first. In early 2-cell, after A-485 treatment, many genes were down-regulated (n=1,490) while very few genes were up-regulated (n=83), suggesting A-485 treatment would mainly affect early 2C minor ZGA genes expression but not maternal RNA decay. To further demonstrate the important role of CBP/p300 for minor ZGA, we performed RNA-seq of zygote after CBP/p300 inhibition (Fig. 4). These results suggested a direct effect of CBP/p300 on minor ZGA. Furthermore, we performed RNA-seq of late 2-cell after CBP/p300 inhibition. At late 2-cell stage, many more genes were down-regulated or up-regulated (Fig. 4E) for major ZGA (Fig. 4F) and maternal RNA (Fig. 4G).

Minor Comments:

1. Please provide row numbers in your manuscript upon resubmission.

We thank the reviewer for the suggestion. We added the row numbers.

2. Typo abstract: "Specifically, CBP/p300 deposits broad H3K27ac domains in zygotes and opens condensed chromatin at putative enhancers"

We thank the reviewer for pointing this out. We corrected the typo.

3. Figure EV1: Please provide info on the abbreviation 'FGO', and specify if the 'public FGO' data that was used by Dahl et al., 2016 is biologically different to the 'GV oocyte' data that as used in this study. Are these the same stages, I assume so?

We added explanation of FGO in the legends. They are the same stages.

4. Typo Page 4: Depletion of HDAC1/2 results in early embryonic lethality. [...] how their activities contribute to ZGA remain largely unknown. Typo Page 10: ATAC-seq peaks that were lost after CBP/p300 inhibition (2x on page). Typo Figure EV1C: Domain bases distribution

We thank the reviewer for pointing out the typo, which we have corrected in the revision.

5. Methods: Please provide at least a few details about the immunostaining in the methods section (permeabilization and fixation buffer, length etc) instead of just giving a reference.

We have added detailed description of the whole-mount immunostaining in the Methods section.

Referee #3:

Histone H3K27ac is associated with the establishment of enhancers/promoters to activate transcription. Wang & Chen et al. used CUT&RUN to reveal the allelic H3K27ac landscape during the maternal-to-zygotic transition. The results suggest that p300/CBP-mediated H3K27ac is required for ZGA and early embryonic development. HDAC activity prevents premature expression of developmental genes. In general, this is a strong manuscript with well designed experiments. However, some important claims are not fully supported by the data and would be strengthened by additional data and analysis as detailed below.

We thank the reviewer for nicely summary and the overall positive comments on our manuscript.

Another weakness is that the results rely entirely on chemical inhibition and have not been corroborated by other perturbations.

We agree with the reviewer that one limitation of our current study is that it relies on inhibitors. Given that CBP/p300 is abundant in oocytes and its rapid dynamics in early embryos, knockdown of CBP/p300 RNA or depletion of the proteins would not be a suitable approach to study its timely role in early embryo development. In addition, given its key role in mediating transcription initiation, it is highly likely that maternal conditional knockout of CBP/p300 would affect oogenesis. Thus, using inhibitors is currently an effective way to study CBP/p300 function in early embryos. We discussed the limitation of our study in the Discussion section.

Major comments:

1. Transient inhibition of CBP/p300 by A-485 from 4-20 hpf caused a 2-cell arrest and downregulation of early 2C ZGA genes (Fig. 4). Since minor ZGA is essential for subsequent major ZGA and development beyond 2-cell stage (Abe et al., 2018, PNAS), an alternative interpretation of this result is that H3K27ac regulates expression of minor ZGA genes and the failure to activate these affects 2C ZGA genes. To distinguish between these possibilities, RNA-seq of zygotes treated with A-485 needs to be performed.

We totally agree with the reviewer. There are two waves of ZGA in mouse embryos – minor ZGA that takes place at late 1-cell to early 2-cell stage and major ZGA that takes place at late 2-cell stage. To dissect the fine scale impact of CBP/p300 inhibition, we further separated minor ZGA genes into zygote minor ZGA genes (n = 30, Dataset EV1) and early 2-cell minor ZGA genes (n = 558, Dataset EV1). Since the zygote minor ZGA genes are very few, we analyzed the early 2-cell first and demonstrated that the early 2C minor ZGA gene expression requires CBP/p300 activity. Following the reviewer's suggestion, we performed RNA-seq of zygote under CBP/p300 inhibition and demonstrated that the CBP/p300 activity is required for the zygote minor ZGA genes (Fig. 4E and F). Together these results suggested a direct effect of CBP/p300 on minor ZGA.

In addition, we performed RNA-seq of late 2-cell after CBP/p300 inhibition. In late 2-cell, both major ZGA genes and maternal RNA decay were affected (Fig. 4F and G). Given that minor ZGA is required for major ZGA (PMID: 29967139), the extensive expression changes in late 2-cell are probably due to the minor ZGA failure. We added description of these results from line 280 to line 302.

2. Abe et al., 2018 also showed DRB treatment from minor ZGA showed loss of H3K4me3 in 2-cell embryos. It is thus necessary to test whether the canonical H3K27ac pattern in 2-cell embryos is dependent on minor ZGA.

Following the reviewer's suggestion, we used DRB to inhibit the minor ZGA then performed H3K27ac and H3K4me3 immunostaining at zygote (8 hpi) and 2-cell (28 hpi) stages (Fig. R2). Consistent with prior report (PMID: 29967139), decrease of H3K4me3 immunostaining signal from 1-cell to 2-cell was not observed in DRB-treated embryos. However, unlike H3K4me3, the H3K27ac level is only mildly affected in DRB treated embryos, suggesting the canonical H3K27ac pattern in 2-cell embryos is largely independent of minor ZGA.

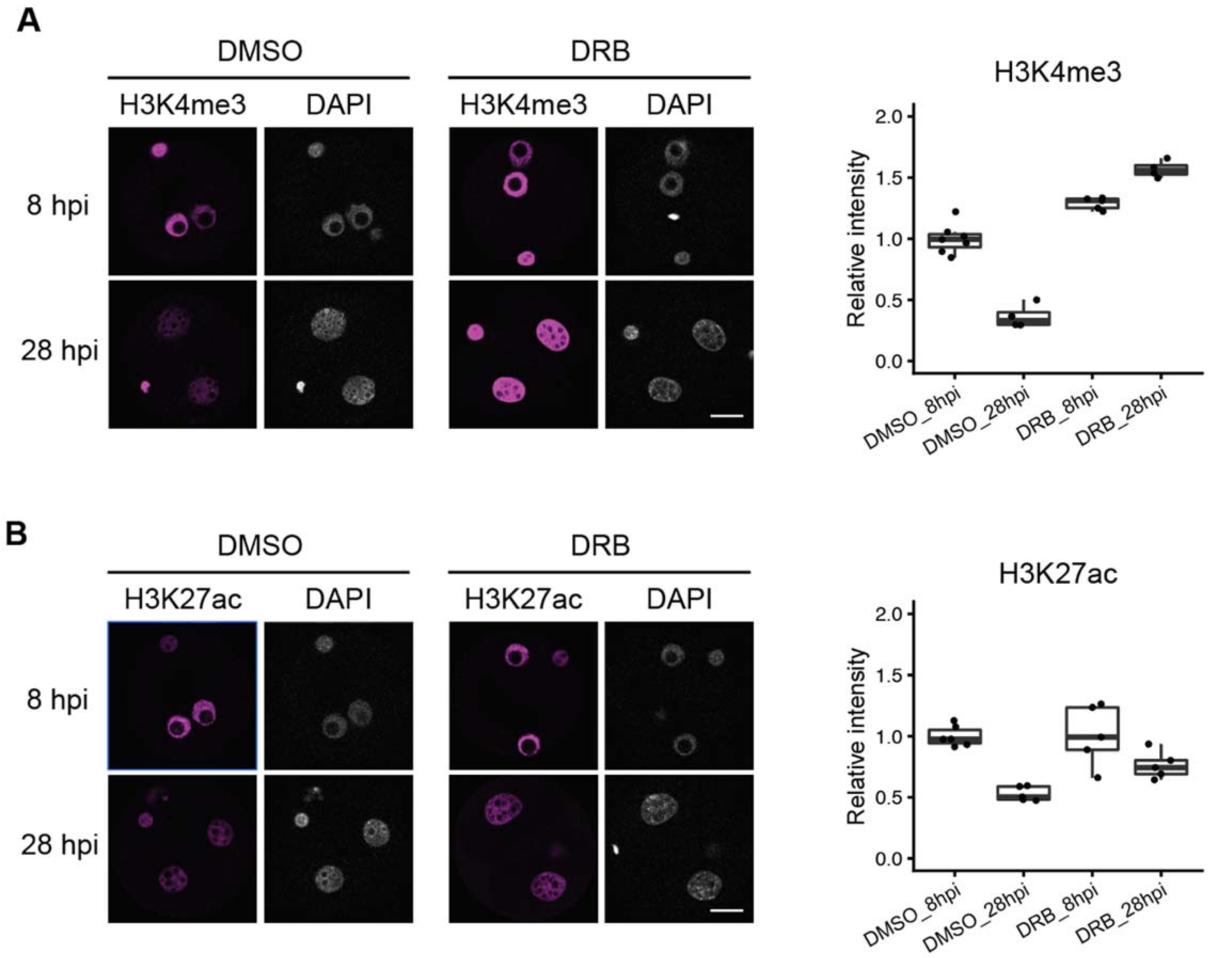

Figure R2. Immunostaining of H3K4me3 (**A**) and H3K27ac (**B**) in zygote (8 hpi) and 2-cell (28 hpi) embryos treated with DMSO or DRB (4-20 hpi, 80 μM). Scale bar: 20 μm.

3. The interpretation of the HDAC inhibitor experiments is complicated by TSA inducing cell cycle arrest via p21 (Hrgovic et al., 2016). The early treatment (0-20 h) might not have any

effect because checkpoints are less active in zygotes. The 8-28 h treatment will overlap with G1 phase of 2C embryos. It is therefore important to delineate the timing of arrest, i.e. does it occur in G1 or G2 phase? If arrest occurs in G1 phase and major ZGA typically occurs during and after S phase, then how can the authors disentangle a direct from an indirect effect on ZGA transcription?

Hrgovic *et al*. showed that 400 nM TSA treatment causes cell cycle arrest, while we used a much lower concentration of TSA treatment (50 nM). In our results, after TSA treatment from 8-28 hpi (2-cell stage), many embryos can still develop into 4-cell stage and mostly arrest at morula stage (Fig. 6D), suggesting that the cell cycle was not affected.

To directly check whether cell cycle was affected in 2-cell under 50 nM TSA treatment, we performed BrdU incorporation assay (Fig. R3). We found that the TSA treated 2-cell embryos were able to enter S phase and G2 phase, demonstrating the cell cycle was not affected under our experimental conditions.

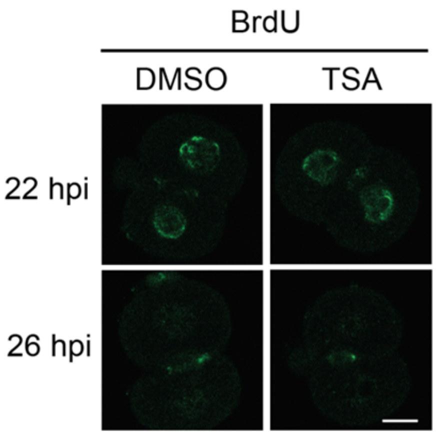

Figure R3. BrdU incorporation assay for 2-cell embryos at S phase (22 hpi) and G2 phase (26 hpi) in control condition (DMSO) and TSA treatment condition. Scale bar: 20 µm.

4. The observation that H3K27 is transiently hypoacetylated in MII oocytes both by immunofluorescent staining and to some extent CUT&RUN is interesting. To exclude that the H3K27ac domain decrease (Fig. 1C) is not due to chromatin inaccessibility for Tn5 in

condensed chromosomes, CUT&RUN should be performed with another histone modification such as H3K9ac or H3S10ph to demonstrate that histone PTMs are readily detectable at this stage using this method.

We totally understand this concern. We had performed CUT&RUN of H3K27me3 and H2AK119ub1 in MII oocytes using the same protocol and the same reagents (different antibodies) and showed good results (PMID: 33821005). In fact, CUT&RUN utilizes MNase which is less affected by chromatin accessibility, whereas methods like CUT&Tag etc. that rely on Tn5 would be largely affected by chromatin accessibility, as systematically evaluated by us previously (https://doi.org/10.1101/2021.07.09.451758).

5. There appears to be hardly any detectable H3K27ac by immunostaining of MII oocytes (Fig. 1A) yet CUT&RUN profiling of the same stage oocytes shows a clear enrichment of H327ac (Fig. 1B). The authors should clarify this apparent discrepancy.

In Fig. 1B the MII oocytes show H3K27ac signals but in fact the signals are very sparse. We chose to display a large region (>1Mb) in Fig. 1B to show the noncanonical broad H3K27ac signals in GV oocytes and zygotes. When displaying a large region, the genome browser would aggregate signals across the region thus it appears to have H3K27ac signals across the region. However, when zooming-in, the MII H3K27ac signals would be very sparse. The domain size distributions called from the CUT&RUN data (Fig. 1C) are consistent with the IF signals in Fig. 1A. We provided our raw data and processed bigwig signals data in GEO under accession number GSE207222.

6. It has recently been reported that H3K27ac is not essential for gene derepression and de novo activation of genes during some cell fate decisions (Sankar et al., Nature 2022) and that PTMs other than H2K27ac are involved in mediating CBP/p300 function. The authors should discuss their results in light of this key finding.

We thank the reviewer for the suggestion. We discussed this from line 449 to line 458.

7. How were ZGA genes defined and what percent of these are significantly downregulated upon CBP/p300 inhibition?

We studied 3 sets of ZGA genes (provided in Dataset EV1): 1) zygote minor ZGA genes (n = 30), 2) early 2C minor ZGA genes (n = 558) and 3) major ZGA genes in late 2C (n = 2,773). The number of genes down-regulated after CBP/p300 inhibition for each set was shown in Figure 4F. The definition of the ZGA genes sets was described in Methods (from line 643 to line 655).

8. The interpretation of all experiments using A-485 critically depends on the specificity of the inhibitor. What is the evidence that A-485 is specific at the concentration used to treat embryos?

The A-485 has been demonstrated as a highly specific and potent inhibitor of CBP/p300 over other HATs and histone methyltransferases in cells, in the original paper that discovered the A-485 (PMID: 28953875). To briefly test its specificity in embryos, we performed immunostaining of H4K16ac and H3K14ac after A-485 treatment in zygote (Fig. R4). Consistent with the fact that H4K16ac and H3K14ac are not deposited by CBP/p300 (PMID: 35042977), immunostaining signals of both histone modifications were not affected by A-485 treatment. In addition, as we have mentioned at the beginning, one limitation of our study is relying on the inhibitors, but for studying CBP/p300 in early embryos, inhibitor is an effective choice currently.

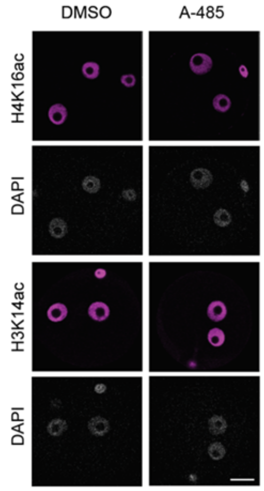

Figure R4. Immunostaining of H4K16ac and H3K14ac in zygote (7 hpi) treated with DMSO or A-485 (4-7 hpi, 10μM). Scale bar: 20 μm.

9. Is there any correlation between the 416 downregulated ZGA genes in A-485 treated embryos (Fig. 4E) and those that have enriched contacts with CBP/p300-dependent open chromatin (Fig. 5E)?

Based on the Hi-C data, we showed these putative enhancers have higher than expected contacts with major ZGA genes. Previously we showed the early 2C minor ZGA genes are down-regulated upon CBP/p300 inhibition (Fig. 4). Thus, we believe major ZGA genes would also be down-regulated. To directly test this, we performed RNA-seq of late 2-cell after CBP/p300 inhibition and examined how major ZGA genes would change. Indeed, most major ZGA genes are down-regulated after CBP/p300 inhibition (Fig. 4F). Thus, we demonstrated that the activity of CBP/p300 is critical for minor ZGA, and for opening the distal chromatin regions at early 2-cell (Fig. 4 and 5), both of which are important for major ZGA. The minor ZGA is known to regulate major ZGA (PMID: 29967139). In this study we showed the CBP/p300-dependent distal opening elements may function as enhancers to regulate the major ZGA genes.

10. A pile-up Hi-C analysis of CBP/p300-dependent distal open chromatin regions and shuffled sets of non-ZGA genes should be performed as a control to determine whether the higher contacts are specific to ZGA genes.

Previously we used the PcG target genes as a control to show the CBP/p300-dependent distal open chromatin regions have higher contacts to major ZGA genes but not PcG target genes (Fig. 5E). Following the reviewer's suggestion, we performed the pile-up analysis with randomly selected non-ZGA genes (with matched number and chromosome distribution) as another control. Results showed no higher than expected contacts between the CBP/p300-dependent distal open chromatin regions and these randomly selected non-ZGA genes promoters (Fig. EV5F).

11. If H3K27ac is priming major ZGA genes, then one would expect that H3K27ac is also enriched at TSS of 2C embryos, yet the signal appears to be lower (Fig. 1F). How can this be explained?

In 2-cell embryos, the exact TSS regions of ZGA genes show lower H3K27ac signals could be due to that the TSSs of ZGA genes are very open and depleted of nucleosomes (Fig. R5).

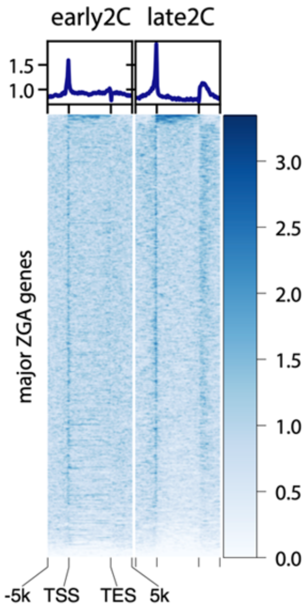

Figure R5. ATAC-seq signals around major ZGA genes in early 2-cell and late 2-cell embryos. The ATAC-seq data were from GEO with accession GSE66390.

Additional comments:

1. The H3K27ac signal is clearly stronger in the nuclei of zygotes compared to 2C embryos (Fig 1A). However, it is puzzling to see that a similar decrease is visible for the polar body, raising a possible concern that the immunofluorescent detection was overall less effective in 2C embryos compared to zygotes.

To test whether the efficiency of immunofluorescence detection is different between 1-cell and 2-cell embryos, we performed co-immunostaining of H3K27me3 and H3K27ac in 1-cell and 2-

cell embryos (Fig. R6). Results showed that the H3K27me3 signal at 2-cell stage was comparable to 1-cell, indicating the immunostaining detection efficiency should be similar between the two developmental stages.

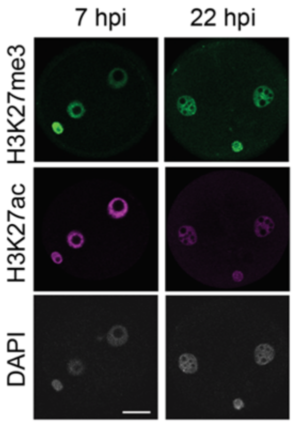

Figure R6. Co-immunostaining of H3K27me3 and H3K27ac in 1-cell (7 hpi) and 2-cell (22 hpi) embryos. Scale bar: 20 µm.

2. Previous work showing that p300 perturbation affects H3K27me3 more at enhancers than promoters (Martire et al., 2020) be referenced.

We thank the reviewer for pointing this out and we have added this paper to the references.

We thank all the reviewers again for their insightful comments which help improve our manuscript tremendously. We hope that the reviewers will be satisfied with our response.

Sincerely,

Yi Zhang, Ph.D
Investigator, HHMI
Professor, BCH & HMS

Thank you for submitting your revised manuscript. We have now received comments from the initial three referees (please see below) and I am pleased to say that they all find that their comments have been addressed and now support publication. Therefore, I would ask you to please resolve a number of editorial issues that are listed in detail below. If you have any further questions regarding the revision or if any of the specific points are unclear, please contact us. Once these final issues are resolved, we will be happy to formally accept the study.

Referee #1:

[no additional comments]

Referee #2:

The authors have addressed my concerns.

Referee #3:

The authors have addressed many of the referees' comments in a satisfactory manner. I support the publication of this work.

I am glad to learn that all the three reviewers support the publication of our manuscript. Thank you very much for organizing the constructive review process which helped improve our manuscript tremendously.

We have resolved all the editorial comments and highlighted the changes in the main text.

2nd Revision - Editorial Decision 23rd Sep 2022

Thank you again for submitting the final revised version of your manuscript. I am happy to inform you that we have now formally accepted the study for publication in The EMBO Journal.

EMBO Press Author Checklist

Corresponding Author Name: Yi Zhang	
Journal Submitted to: The EMBO Journal	
Manuscript Number: EMBOJ-2022-112012	

USEFUL LINKS FOR COMPLETING THIS FORM
The EMBO Journal - Author Guidelines
EMBO Reports - Author Guidelines
Molecular Systems Biology - Author Guidelines
EMBO Molecular Medicine - Author Guidelines

Reporting Checklist for Life Science Articles (updated January
This checklist is adapted from Materials Design Analysis Reporting (MDAR) Checklist for Authors. MDAR establishes a minimum set of requirements in transparent reporting in the life sciences (see Statement of Task: 10.31222/osf.io/9sm4x). Please follow the journal's guidelines in preparing your
Please note that a copy of this checklist will be published alongside your article.

Abridged guidelines for figures
1. Data
The data shown in figures should satisfy the following conditions:
- the data were obtained and processed according to the field's best practice and are presented to reflect the results of the experiments in an accurate and unbiased manner.
- ideally, figure panels should include only measurements that are directly comparable to each other and obtained with the same assay.
- plots include clearly labeled error bars for independent experiments and sample sizes. Unless justified, error bars should not be shown for technical
- if n<5, the individual data points from each experiment should be plotted. Any statistical test employed should be justified.
- Source Data should be included to report the data underlying figures according to the guidelines set out in the authorship guidelines on Data

2. Captions
Each figure caption should contain the following information, for each panel where they are relevant:
- a specification of the experimental system investigated (eg cell line, species name).
- the assay(s) and method(s) used to carry out the reported observations and measurements.
- an explicit mention of the biological and chemical entity(ies) that are being measured.
- an explicit mention of the biological and chemical entity(ies) that are altered/varied/perturbed in a controlled manner.
- the exact sample size (n) for each experimental group/condition, given as a number, not a range;
- a description of the sample collection allowing the reader to understand whether the samples represent technical or biological replicates (including how many animals, litters, cultures, etc.).
- a statement of how many times the experiment shown was independently replicated in the laboratory.
- definitions of statistical methods and measures:
  - common tests, such as t-test (please specify whether paired vs. unpaired), simple χ2 tests, Wilcoxon and Mann-Whitney tests, can be unambiguously identified by name only, but more complex techniques should be described in the methods section;
  - are tests one-sided or two-sided?
  - are there adjustments for multiple comparisons?
  - exact statistical test results, e.g., P values = x but not P values < x;
  - definition of 'center values' as median or average;
  - definition of error bars as s.d. or s.e.m.

Please complete ALL of the questions below.
Select "Not Applicable" only when the requested information is not relevant for your study.

Materials

**Newly Created Materials**	**Information included in the manuscript?**	**In which section is the information available?** (Reagents and Tools Table, Materials and Methods, Figures, Data Availability Section)
New materials and reagents need to be available; do any restrictions apply?	Not Applicable	

**Antibodies**	**Information included in the manuscript?**	**In which section is the information available?** (Reagents and Tools Table, Materials and Methods, Figures, Data Availability Section)
For **antibodies** provide the following information:<br>- Commercial antibodies: RRID (if possible) or supplier name, catalogue number and or/clone number<br>- Non-commercial: RRID or citation	Yes	Materials and Methods

**DNA and RNA sequences**	**Information included in the manuscript?**	**In which section is the information available?** (Reagents and Tools Table, Materials and Methods, Figures, Data Availability Section)
**Short novel DNA or RNA including primers, probes**: provide the sequences.	Not Applicable	

**Cell materials**	**Information included in the manuscript?**	**In which section is the information available?** (Reagents and Tools Table, Materials and Methods, Figures, Data Availability Section)
**Cell lines:** Provide species information, strain. Provide accession number in repository **OR** supplier name, catalog number, clone number, and/**OR** RRID.	Not Applicable	
**Primary cultures:** Provide species, strain, sex of origin, genetic modification status.	Not Applicable	
Report if the cell lines were recently **authenticated** (e.g., by STR profiling) and tested for mycoplasma contamination.	Not Applicable	

**Experimental animals**	**Information included in the manuscript?**	**In which section is the information available?** (Reagents and Tools Table, Materials and Methods, Figures, Data Availability Section)
**Laboratory animals or Model organisms:** Provide species, strain, sex, age, genetic modification status. Provide accession number in repository **OR** supplier name, catalog number, clone number, **OR** RRID.	Yes	Materials and Methods
**Animal observed in or captured from the field:** Provide species, sex, and age where possible.	Not Applicable	
Please detail **housing and husbandry conditions**.	Yes	Materials and Methods

**Plants and microbes**	**Information included in the manuscript?**	**In which section is the information available?** (Reagents and Tools Table, Materials and Methods, Figures, Data Availability Section)
**Plants:** provide species and strain, ecotype and cultivar where relevant, unique accession number if available, and source (including location for collected wild specimens).	Not Applicable	
**Microbes:** provide species and strain, unique accession number if available, and source.	Not Applicable	

**Human research participants**	**Information included in the manuscript?**	**In which section is the information available?** (Reagents and Tools Table, Materials and Methods, Figures, Data Availability Section)
If collected and within the bounds of privacy constraints report on age, sex and gender or ethnicity for all study participants.	Not Applicable	

**Core facilities**	**Information included in the manuscript?**	**In which section is the information available?** (Reagents and Tools Table, Materials and Methods, Figures, Data Availability Section)
If your work benefited from core facilities, was their service mentioned in the acknowledgments section?	Not Applicable	

Design

Study protocol	Information included in the manuscript?	In which section is the information available? (Reagents and Tools Table, Materials and Methods, Figures, Data Availability Section)
If study protocol has been **pre-registered, provide DOI in the manuscript**. For clinical trials, provide the trial registration number **OR** cite DOI.	Not Applicable	
Report the **clinical trial registration number** (at ClinicalTrials.gov or equivalent), where applicable.	Not Applicable	

Laboratory protocol	Information included in the manuscript?	In which section is the information available? (Reagents and Tools Table, Materials and Methods, Figures, Data Availability Section)
Provide DOI OR other citation details if **external detailed step-by-step protocols** are available.	Yes	Materials and Methods

Experimental study design and statistics	Information included in the manuscript?	In which section is the information available? (Reagents and Tools Table, Materials and Methods, Figures, Data Availability Section)
Include a statement about **sample size** estimate even if no statistical methods were used.	Not Applicable	
Were any steps taken to minimize the effects of subjective bias when allocating animals/samples to treatment (e.g. **randomization procedure**)? If yes, have they been described?	Not Applicable	
Include a statement about **blinding** even if no blinding was done.	Not Applicable	
Describe **inclusion/exclusion criteria** if samples or animals were excluded from the analysis. Were the criteria pre-established?<br><br>If sample or data points were omitted from analysis, report if this was due to attrition or intentional exclusion and provide justification.	Not Applicable	
For every figure, are **statistical tests** justified as appropriate? Do the data meet the assumptions of the tests (e.g., normal distribution)? Describe any methods used to assess it. Is there an estimate of variation within each group of data? Is the variance similar between the groups that are being statistically compared?	Yes	Figure legends

Sample definition and in-laboratory replication	Information included in the manuscript?	In which section is the information available? (Reagents and Tools Table, Materials and Methods, Figures, Data Availability Section)
In the figure legends: state number of times the experiment was **replicated** in laboratory.	Yes	Figure legends
In the figure legends: define whether data describe **technical or biological replicates**.	Yes	Figure legends

Ethics

Ethics	Information included in the manuscript?	In which section is the information available? (Reagents and Tools Table, Materials and Methods, Figures, Data Availability Section)
Studies involving **human participants**: State details of **authority granting ethics approval** (IRB or equivalent committee(s), provide reference number for approval.	Not Applicable	
Studies involving **human participants**: Include a statement confirming that **informed consent** was obtained from all subjects and that the experiments conformed to the principles set out in the WMA Declaration of Helsinki and the Department of Health and Human Services Belmont Report.	Not Applicable	
Studies involving **human participants:** For publication of **patient photos**, include a statement confirming that consent to publish was obtained.	Not Applicable	
Studies involving experimental **animals**: State details of **authority granting ethics approva**l (IRB or equivalent committee(s), provide reference number for approval. Include a statement of compliance with ethical regulations.	Yes	Materials and Methods
Studies involving **specimen and field samples:** State if relevant **permits** obtained, provide details of authority approving study; if none were required, explain why.	Not Applicable	

Dual Use Research of Concern (DURC)	Information included in the manuscript?	In which section is the information available? (Reagents and Tools Table, Materials and Methods, Figures, Data Availability Section)
Could your study fall under dual use research restrictions? Please check biosecurity documents and list of **select agents and toxins** (CDC): https://www.selectagents.gov/sat/list.htm	Not Applicable	
If you used a select agent, is the security level of the lab appropriate and reported in the manuscript?	Not Applicable	
If a study is subject to dual use research of concern regulations, is the name of the **authority granting approval and reference number** for the regulatory approval provided in the manuscript?	Not Applicable	

Reporting

The MDAR framework recommends adoption of discipline-specific guidelines, established and endorsed through community initiatives. Journals have their own policy about requiring specific guidelines and recommendations to complement MDAR.

Adherence to community standards	Information included in the manuscript?	In which section is the information available? (Reagents and Tools Table, Materials and Methods, Figures, Data Availability Section)
State if relevant guidelines or checklists (e.g., **ICMJE, MIBBI, ARRIVE, PRISMA**) have been followed or provided.	Not Applicable	
For **tumor marker prognostic studies,** we recommend that you follow the **REMARK** reporting guidelines (see link list at top right). See author guidelines, under 'Reporting Guidelines'. Please confirm you have followed these guidelines.	Not Applicable	
For **phase II and III randomized controlled trials**, please refer to the **CONSORT** flow diagram (see link list at top right) and submit the CONSORT checklist (see link list at top right) with your submission. See author guidelines, under 'Reporting Guidelines'. Please confirm you have submitted this list.	Not Applicable	

Data Availability

Data availability	Information included in the manuscript?	In which section is the information available? (Reagents and Tools Table, Materials and Methods, Figures, Data Availability Section)
Have **primary datasets** been deposited according to the journal's guidelines (see 'Data Deposition' section) and the respective accession numbers provided in the Data Availability Section?	Yes	Data Availability
Were **human clinical and genomic datasets** deposited in a public access-controlled repository in accordance to ethical obligations to the patients and to the applicable consent agreement?	Not Applicable	
Are **computational models** that are central and integral to a study available without restrictions in a machine-readable form? Were the relevant accession numbers or links provided?	Not Applicable	
If publicly available data were reused, provide the respective **data citations in the reference list**.	Yes	Table EV1
